# Selective Histone Deacetylase 6 Inhibitors Restore Cone Photoreceptor Vision or Outer Segment Morphology in Zebrafish and Mouse Models of Retinal Blindness

**DOI:** 10.3389/fcell.2020.00689

**Published:** 2020-08-26

**Authors:** Husvinee Sundaramurthi, Sarah L. Roche, Guinevere L. Grice, Ailis Moran, Eugene T. Dillion, Giuseppe Campiani, James A. Nathan, Breandán N. Kennedy

**Affiliations:** ^1^UCD Conway Institute, University College Dublin, Dublin, Ireland; ^2^UCD School of Biomolecular and Biomedical Science, University College Dublin, Dublin, Ireland; ^3^Systems Biology Ireland, University College Dublin, Dublin, Ireland; ^4^UCD School of Medicine, University College Dublin, Dublin, Ireland; ^5^School of Biochemistry, University College Cork, Cork, Ireland; ^6^Cambridge Institute of Therapeutic Immunology & Infectious Disease, University of Cambridge, Cambridge, United Kingdom; ^7^Mass Spectrometry Resource, UCD Conway Institute, University College Dublin, Dublin, Ireland; ^8^Department of Biotechnology, Chemistry and Pharmacy, Department of Excellence, University of Siena, Siena, Italy

**Keywords:** HDAC6 inhibitors, tubastatin A, retinal degenerations, Hif-1α, proteome profiling

## Abstract

Blindness arising from retinal or macular degeneration results in significant social, health and economic burden. While approved treatments exist for neovascular (‘*wet’*) age-related macular degeneration, new therapeutic targets/interventions are needed for the more prevalent atrophic (‘*dry’*) form of age-related macular degeneration. Similarly, in inherited retinal diseases, most patients have no access to an effective treatment. Although macular and retinal degenerations are genetically and clinically distinct, common pathological hallmarks can include photoreceptor degeneration, retinal pigment epithelium atrophy, oxidative stress, hypoxia and defective autophagy. Here, we evaluated the potential of selective histone deacetylase 6 inhibitors to preserve retinal morphology or restore vision in zebrafish *atp6v0e1*^–/–^ and mouse *rd10* models. Histone deacetylase 6 inhibitor, tubastatin A-treated *atp6v0e1*^–/–^ zebrafish show marked improvement in photoreceptor outer segment area (44.7%, p = 0.027) and significant improvement in vision (8-fold, *p* ≤ 0.0001). Tubastatin A-treated *rd10/rd10* retinal explants show a significantly (*p* = 0.016) increased number of outer-segment labeled cone photoreceptors. *In vitro*, ATP6V0E1 regulated HIF-1α activity, but significant regulation of HIF-1α by histone deacetylase 6 inhibition in the retina was not detected. Proteomic profiling identified ubiquitin-proteasome, phototransduction, metabolism and phagosome as pathways, whose altered expression correlated with histone deacetylase 6 inhibitor mediated restoration of vision.

## Introduction

Inherited retinal diseases (IRD) and age-related macular degenerations (AMD) are heterogenous groups of diseases causing blindness. With a prevalence of 1 in 3000 people affected worldwide, common forms of IRD include retinitis pigmentosa (RP), cone/rod dystrophy, Leber congenital amaurosis and Stargardt disease (Duncan et al., 2018). Variants in over 270 genes can result in monogenic IRD, this heterogeneity makes clinical diagnosis and prognosis challenging (Daiger et al., 1998). A significant milestone was the recent regulatory approval of Luxturna^®^, a gene therapy for Leber congenital amaurosis patients with recessive mutations in RPE65 (Duncan et al., 2018; Gordon et al., 2019). Neovascular (‘*wet’*) and atrophic (‘*dry’*) AMD have a global prevalence of 170 million. Anti-vascular endothelial growth factor therapies are approved treatments for wet-AMD (Hernandez-Zimbron et al., 2018; Holekamp, 2019). However, there is an unmet clinical need for treatments for dry-AMD (Pennington and DeAngelis, 2016; Hernandez-Zimbron et al., 2018). Drug-based (*e.g.*, neuroprotectants, anti-inflammatory, statins, visual cycle inhibitors, complement inhibitors) clinical trials for IRD and AMD are on-going, albeit many of the previous ones demonstrated limited success (Waugh et al., 2018; Sundaramurthi et al., 2019). This highlights that the vast majority of IRD and dry-AMD patients currently have no access to an effective treatment.

Histone deacetylases (HDACs) are enzymes that regulate gene expression through the removal of an acetyl group from histone and non-histone proteins (de Ruijter et al., 2003). There are 18 mammalian HDACs, classified into four main categories in accordance to their homology with related proteins in yeast (Xu et al., 2007; Haberland et al., 2009). Class I comprises of HDACs 1, 2, 3, and 8, they localize to the nucleus and are dependent on Zn^2+^ for their activity. Class II are also Zn^2+^ dependent, and functional in both the nucleus and cytoplasm. The roles of Class II HDACs are more tissue-specific, and they are sub-divided into IIa (HDACs 4, 5, 7, and 9) and IIb (HDACs 6 and 10) (Yang and Gregoire, 2005). Sirtuins or Class III HDACs (SIRT 1-7) are NAD^+^-dependent and are found in the nucleus, cytoplasm or mitochondria (Park and Kim, 2020). Not much is known about Class IV, which is made up of only HDAC11. Histone deacetylase inhibitors (HDACi) are gaining attention as potential therapeutic agents for various diseases, including cancer, neurodegenerative diseases and inflammatory diseases (Zhang et al., 2015; Hull et al., 2016; Eckschlager et al., 2017; Ziemka-Nalecz et al., 2018).

Histone deacetylase inhibitors exert neuroprotective action in central nervous system diseases *e.g.*, Parkinson’s, Alzheimer’s, amyotrophic lateral sclerosis and spinal muscular atrophy (Lai et al., 2017; Janczura et al., 2018; Thomas and D’Mello, 2018). Preclinical studies show pan-HDAC inhibition to efficiently restore visual function in zebrafish and mouse models of inherited blindness (Trifunovic et al., 2016; Daly et al., 2017; Ratay et al., 2018; Trifunovic et al., 2018). In contrast, in *Xenopus* RP models, pan-HDAC inhibition resulted in beneficial or unfavorable effects depending on the rhodopsin mutation tested (Vent-Schmidt et al., 2017). Similarly, clinical trials evaluating pan-HDACi were contradictory, improving visual acuity in some RP patients while detrimental to vision in others (Clemson et al., 2011; van Schooneveld et al., 2011; Sisk, 2012; Bhalla et al., 2013; Iraha et al., 2016; Totan et al., 2017). Due to these controversies, we investigated if selective inhibitors of the HDAC6 isoform offer an effective alternative to restore or preserve vision and retinal morphology.

Unlike other HDACs, HDAC6 is a cytoplasmic protein with two catalytic domains, which deacetylates non-histone proteins (Boyault et al., 2007). HDAC6 modulates proteins regulating numerous cellular pathways including the ubiquitin-proteasome, autophagy, stress responses (*e.g.*, heat-shock response), redox signaling, inflammation and intracellular trafficking (Boyault et al., 2007; Li et al., 2013; Richter-Landsberg and Leyk, 2013; Simoes-Pires et al., 2013; Ryu et al., 2017). Several studies report beneficial effects mediated by HDAC6 inhibitors (HDAC6i) in neurodegenerative diseases (Benoy et al., 2017; Cosenza and Pozzi, 2018). Selective HDAC6i rectified mitochondrial transport defects in Charcot-Marie-Tooth disease models, and reduced hyperphosphorylated tau levels and amyloid-beta accumulation in Alzheimer’s models in mice (Simoes-Pires et al., 2013; Zhang et al., 2014; Benoy et al., 2017). The selective HDAC6i, tubastatin A (TubA), was neuroprotective to photoreceptors subjected to oxidative stress and restored vision in the *atp6v0e1*^–/–^ zebrafish model of inherited blindness (Leyk et al., 2017). This zebrafish line harbors a mutation in the vacuolar H^+^ ATPase (V-ATPase), V0 subunit e1 gene (*atp6v0e1*), and presents with retinal degeneration, hypopigmentation, pericardial/abdominal edema and a deflated swim bladder (Daly et al., 2017). V-ATPases are ATP-dependent, multi-subunit proton pump complexes that acidify cellular organelles (*e.g.*, lysosomes, phagosomes and endosomes) and centrally linked to the process of autophagy (Pamarthy et al., 2018). Notably, dysregulated autophagy is reported in models of IRD and AMD (Mitter et al., 2014; Moreno et al., 2018). Mutations or deficiencies in V-ATPases and accessory proteins cause frontotemporal lobar degeneration, Alzheimer’s, a rare form of Parkinson’s, osteopetrosis or osteoporosis (Lee et al., 2015; Colacurcio and Nixon, 2016; Duan et al., 2018). In the eye, the V-ATPase accessory subunit TMEM199 is genetically linked with susceptibility to developing AMD, suggesting potential roles of other V-ATPase subunits and accessory subunits in contributing to the pathogenesis of AMD (Ratnapriya et al., 2019). In RPE lysosomes, the lipofuscin fluorophore A2-E inhibits the V-ATPase pump which may contribute to AMD pathogenesis by impairing phagolysosomal degradation of phagocytosed photoreceptor outer segment phospholipids (Finnemann et al., 2002; Bergmann et al., 2004). Impaired function of numerous V-ATPase subunits, *atp6v1h*, *atp6v1f*, *atp6v1e1*, *atp6v0c*, and *atp6v0d1*, and accessory protein, *atp6ap1*, results in inherited blindness and retinal degeneration in zebrafish, and other pre-clinical models (Nuckels et al., 2009; Kawamura et al., 2010; Shine et al., 2014).

A hallmark of IRD is oxidative damage leading to a hypoxic environment and stress in the retina (Masuda et al., 2017; Trachsel-Moncho et al., 2018). Recently, it was reported that V-ATPase subunit, *ATP6V1A1*, and accessory proteins, *TMEM199* and *CCDC115*, regulate HIF-1α levels through controlling free intracellular iron levels (Miles et al., 2017). However, an equivalent role for the *ATP6V0E1* subunit was not investigated. Reducing HIF-1α levels has proven to protect photoreceptor cells from degeneration thus, providing a potential therapeutic target for IRD (Vadlapatla et al., 2013; Barben et al., 2018a; Samardzija et al., 2019). We investigated whether *ATP6V0E1* regulates HIF-1α signaling and whether inhibition of HDAC6 modulates HIF-1α target genes in the retina.

The goal of the present study was to identify HDAC6 inhibitors that elicits robust neuroprotection in genetic model(s) of retinal blindness and to understand the molecular mechanism through which neuroprotection and restoration of vision is mediated. We show TubA is highly effective in restoring visual function and is neuroprotective of cone photoreceptor cells in zebrafish and mice models. TubA regulation of cellular pathways including phototransduction, metabolism, ubiquitin-proteasome and phagocytosis correlated with improvement of cone photoreceptor vision.

## Methodology

### Animal Husbandry and Maintenance

#### Zebrafish

All animal experiments were performed with prior approval granted by UCD Animal Research Ethics Committee and Health Products Regulatory Authority (License AE18982/P062 and AE18982/P134), Ireland. Zebrafish environmental parameters are reported (dx.doi.org/10.17504/protocols.io.8vshw6e) and husbandry was carried out in accordance to established laboratory protocols (Howe et al., 2013). Embryos and larvae from wildtype (Tübingen) and *atp6v0e1*^–/–^ knock-out (official allele designation *atp6v0e1^*UCD*6^)* backgrounds were maintained on a 14 h light/10 h dark cycle at 28.5°C in embryo medium (0.137 M NaCl, 5.5 mM Na_2_HPO_4_, 5.4 mM KCl, 1.3 mM CaCl_2_, 0.44 mM KH_2_PO_4_, 1 mM MgSO_4_ and 4.2 mM NaHCO_3_, with conductivity of 1200 μS and pH 7) with methylene blue (Daly et al., 2017). Adults were maintained in a recirculating water system at 28°C under 14 h light/10 h dark cycle and fed daily with brine shrimp and dry pellet food. Homozygous *atp6v0e1^–/–^* larvae were bred by incrosses of carriers and maintained by incrosses of carriers and outcrosses of carriers to wildtype fish.

#### Mice

All animals were handled and maintained following the ARVO Statement for the Use of Animals in Ophthalmic and Vision Research (License AE19130). Experiments were approved by University College Cork Animal Experimentation Ethics Committee and were performed using both male and female homozygous *rd10* mice (B6.CXB1-Pde6brd10/J). Mice were supplied by the Biological Services Unit, University College Cork and were humanely euthanized by cervical dislocation. Mice were exposed to a 12 h light/12 h dark cycle.

### Drug Treatments in Zebrafish Larvae and *rd10* Rodent Explants

#### Zebrafish Larvae Drug Treatments

Wildtype larvae were treated with increasing drug concentrations(1–100 μM) of tubacin (Sigma-Aldrich, St. Louis, MO, United States), ACY-1215 (Ricolinostat; Selleck Chemicals, Houston, TX, United States), NF2373 (provided by Giuseppe Campiani) and Fe (III) citrate (Sigma-Aldrich). To determine the maximum tolerated concentration (MTC), four 3 dpf larvae were placed in each well of a 48-well plate with either 400 μl of 0.1% DMSO (dimethyl sulfoxide, vehicle control) or select concentrations of chosen drugs in duplicate or triplicate until 5 dpf. The MTC was determined based on the given criteria: (1) an adverse effect was not observed in overall gross morphology of surviving (> 80% survival rate) larvae and (2) the average number of OKR saccades was not reduced by > 15% compared to controls. 12 siblings (*atp6v0e1*^+/+^ and *atp6v0e1*^+/−^ larvae) and *atp6v0e1^–/–^* larvae each were used to test the efficacy of select HDAC6i at the desired MTC. At 3 dpf, larvae were placed in 60 × 15 mm petri dishes and treated with either 10 ml of 100 μM TubA (Selleck Chemicals), 10 μM Tubacin, 10 μM ACY-1215, 25 μM NF2373 or 100 μM Fe (III) citrate + 100 μM ascorbic acid (Sigma-Aldrich) + 1 mg/ml lactoferrin (Sigma-Aldrich) and 0.1% DMSO. All drug solutions were prepared in embryo medium. Larvae in dishes were placed into a secondary container and incubated in a 14 h light/10 h dark cycle at 28.5°C, for 4 h or between 3 – 8 dpf, with or without changes to drug solution.

#### Rodent Retinal Explant Culture

Retinal explants were prepared from postnatal day 14 (P14) *rd10* mice as described previously (Roche et al., 2017, 2018; Ruiz Lopez et al., 2017). P14 to P18 explant culture was selected based on the significantly reduced outer segment length observed in *rd10* retina compared to wildtype retinae from P10 to P15, without a corresponding significant change in outer nuclear layer (ONL) thickness (Roche et al., 2017). Therefore, the selected time frame allows investigation of rescue to rod and cone outer segment morphology at early stages of degeneration. Briefly, eyes were enucleated and transferred to a sterile laminar flow hood. Whole retinae were carefully dissected and placed photoreceptor-side down without the RPE, on a cell culture insert in advanced Dulbecco’s modified Eagle’s medium (DMEM)-F12 (Sigma-Aldrich) supplemented with 1% penicillin/streptomycin. An attached RPE is important for retinal explant cultures from earlier developmental stages to mature, but in P14 explants the murine retina has already elaborated outer segments *in vivo* by P12 (Pinzon-Duarte et al., 2000; Bassett and Wallace, 2012). Retinal explants were cultured for 4 days (96 h) in six-well plates with 1.5 mL media per well/explant, with a media change at day 3. Explants were exposed to various concentrations of TubA (200 mM stock solution in DMSO) ranging from 10 to 100 μM. All explants were exposed to the same volume of DMSO. The vehicle control was exposed to DMSO alone.

### Measure of Visual Function – Optokinetic Response (OKR) Assay

Optokinetic Response assays were performed as described previously (Daly et al., 2017). Vehicle and drug treated larvae were removed from treatment solution and replaced with embryo medium. Larvae were individually immobilized in 9% methylcellulose (prepared in embryo medium), in a 60 × 15 mm petri dish. Each larva was placed in the center of the OKR rig and subjected to rotating black and white stripes (20 vertical, 1 cm thick stripes, angle subtended 33.2°, contrast 99%), rotated at 18 revolutions per minute (RPM), for 30 s in the clockwise and anti-clockwise direction. Saccadic eye movements produced by the larva was counted for a total duration of 1 min.

### Analysis of Retinal Histology

#### Light and Electron Microscopy Imaging

Zebrafish larval samples were euthanized in tricaine and fixed immediately in 4% paraformaldehyde (PFA) and 2.5% glutaraldehyde prepared in 0.1 M Sorensons’s buffer (pH 7.3) overnight up to a week at 4°C. Then samples were post-fixed with 1% osmium tetraoxide and dehydrated with increasing concentrations of ethanol. This was followed by incubation in decreasing concentration of propylene oxide:EPON resin mixtures. Lastly, samples were embedded in 100% EPON resin. Samples in resin were polymerized overnight at 60°C. 500 nM–1 μM thick sections were collected for light microscopy and 80 nM thick sections for electron microscopy using Leica EM UC6 microtome (Leica, Wetzlar, Germany). Sections for light microscopy were mounted on to glass slides and stained with toluidine blue prior to imaging with Leica DMLB bright field illumination microscope and a Leica DFC 480 camera (Leica). Sections collected for electron microscopy were stained with uranyl acetate and lead citrate and imaged with FEI Tecnai 120 transmission electron microscope (Thermo Fisher Scientific, Waltham, MA, United States). Three sections, taken from the central retina per sample, imaged at a magnification of 2550x were used for the measurement of total number of photoreceptors and total area of photoreceptor outer segments. Photoreceptors were counted if both the outer and inner segments were identifiable. The area of individual photoreceptor’s outer segment was measured using ImageJ, by selecting each region of interest and utilizing the Measurement tool. A total of two individual larvae were analyzed per sample group.

#### Immunohistochemistry and Confocal Microscopy

Zebrafish larvae were fixed in 4% PFA overnight at 4°C. Larvae were washed with 1 × PBST for 5 min, 3 times. Larvae were mounted in 1.5% low melting agarose/5% sucrose/1 × PBST and cryoprotected in 30% sucrose/1 × PBST solution, overnight at 4°C. Samples were covered in OCT Compound Tissue-Tek (Sigma-Aldrich) and flash frozen in liquid nitrogen. 16 μM thick sections were prepared in the cryotome (Leica) and alternate sections were collected onto four Superfrost glass slides (Fisher Scientific, Waltham, MA, United States) and stored at −20°C. Sections were dried at 37°C for 1 h and rehydrated with 1 × PBST for 5 mins. Samples were labeled with primary antibodies, zpr-1 (1:100, ZIRC) or zpr-3 (1:100, ZIRC), for 2 h at room temperature (RT). Slides were washed with 1 × PBST, 5 times for 7 min each. Sections were incubated with secondary antibody, Anti mouse-Alexa Fluor^®^ 488 Conjugate (1:500, #4408, Cell Signaling Technology, Danvers, MA, United States) for 1 h at RT. Slides were washed as before and incubated with DAPI (1:500, Sigma-Aldrich) for 15 min, RT. Samples were washed and mounted with Mowiol (Sigma-Aldrich) and stored at 4°C. All samples were imaged at a lens magnification of 20x or 63x (with an additional digital zoom of 3x) using Zeiss LSM 510 laser scanning confocal microscope. Images were taken in the same session to ensure identical microscope settings used when imaging across each treatment groups. Individual cone cells, labeled with zpr-1 staining, were quantified from three sections per sample (*N* = 3). The total area of photoreceptor outer segments (labeled with zpr-3, three sections per sample and *N* = 3) was measured using ImageJ, by selecting each region of interest and utilizing the Measurement tool. Statistical analysis was performed using unpaired Student’s *T* test.

Mice retinal whole mounts were fixed in 4% PFA for 30 mins, at RT. Following washes, retinae to be used for sectioning were cryoprotected in 15% sucrose in 1 × PBS for 1 h, 20% sucrose for 1 h, and 30% sucrose overnight, all at 4°C. Retinae were submerged and frozen in cryochrome (Thermo Fisher Scientific) and sectioned on a cryostat (Leica). Sections (10 μm) were collected on Superfrost glass slides (Fisher Scientific) and stored at −80°C. Sections were blocked and permeabilized with 0.1% Triton X-100 and 5% donkey serum in 1 × PBS for 30 min and incubated with primary antibody diluted in 5% donkey serum overnight at 4°C (Rhodopsin, Thermo Fischer Scientific #MS-1233, 1:100; Cone Arrestin, Merck Millipore #AB15282, 1:100, Burlington, MA, United States). Retinal whole mounts were blocked and permeabilized with 4% Triton X-100 and 5% donkey serum in 1 × PBS for 1.5 hrs and incubated with primary antibody overnight at 4°C (Rhodopsin, Thermo Fischer Scientific #MS-1233, 1:100; Cone Arrestin, Merck Millipore #AB15282, 1:100). Following washes, sections/retinal whole mounts were incubated with secondary antibody (Alexa Fluor donkey anti-mouse/rabbit/goat with either a Alexa Fluor^®^ 488 or a Alexa Fluor^®^ 594 fluorescent probe; Molecular Probes, Eugene, OR, United States) and Hoechst 33342 nuclear stain (1:10,000; Thermo Fischer Scientific) for 1 and 2 h, respectively, at RT. Eliminating the primary antibody in solution served as a negative control. Sections/retinal whole mounts were mounted using Mowiol^®^ (Sigma-Aldrich) with DABCO^®^ antifade agent (Sigma-Aldrich). Retinal sections and whole-mount preparations were viewed using a Leica DM LB2 microscope with Nikon Digital Sight DS-U2 camera (Nikon, Tokyo, Japan), using × 10 and × 40 objectives. Images were taken using the software NIS-Elements version 3.0 (Nikon). All images shown were taken in the central retina. Numbers of nuclei were counted using Hoechst 33342. Rods and cones were identified from rhodopsin- and cone arrestin- positivity, respectively. Fluorescence intensity measurements were taken using ImageJ software (National Institutes of Health, Bethesda, MD, United States). Samples were processed at the same time using the same buffers and antibody solutions. Identical microscope settings were used when imaging each preparation across treatment groups, and images were taken in the same session.

### Whole Genome Sequencing

Genomic DNA was isolated from 5 dpf whole larvae using the DNeasy Blood and Tissue Kit (Qiagen, Hilden, Germany), according to manufacturer’s instructions. A pooled sample of 20 siblings (*atp6v0e1*^+^*^/^*^+^ and *atp6v0e1*^+/−^ larvae) and *atp6v0e1^–/–^* larvae each were used. Samples were outsourced to Novogene Co., Ltd. (Beijing, China) for genome sequencing and bioinformatic analysis with the following parameters: Illumina PE150 sequencer with 30x coverage, mapping of sequence reads with CASAVA software. Integrative Genomics Viewer (IGV) (Robinson et al., 2011; Thorvaldsdottir et al., 2013) software was used to analyze sequence of *atp6v0e1* gene in Chromosome 14, for mutation identification.

### RNA Isolation

Sixty eyes each were dissected from 3 and 6 days old siblings (*atp6v0e1*^+^*^/+^* and *atp6v0e1*^+/−^ larvae) and *atp6v0e1^–/–^* larvae treated with either 100 μM TubA or 0.1% DMSO, and RNA was isolated at two time points, 4 h and 3 days, post treatment. Prior to dissection, drug treated larvae were washed with embryo medium thrice, euthanized in tricaine and placed into RNAlater overnight at 4°C. Dissected eye tissues were homogenized in Lysis buffer provided in the mirVana™ miRNA Isolation Kit (Thermo Fisher Scientific) with a 26 gauge 3/8^II^ needle/syringe. RNA was isolated from the homogenized eye tissues using mirVana™ miRNA Isolation Kit in accordance to manufacturer’s protocol. Eluted RNA samples were precipitated with 100% ethanol and 3M sodium acetate, overnight at −20°C. Samples were spun down for 1 hr at 14,000 RPM at 4°C. The supernatant was removed, and pellet was washed with 80% ethanol as before. Samples were dried, pellet was re-dissolved in ultrapure water and stored at −80°C till use. Sample purity (RNA integrity number) and concentration was measured using the Agilent 2100 Bioanalyzer System (Agilent, Santa Clara, CA, United States).

### Quantification of RNA Transcript Levels Using qRT-PCR

cDNA was synthesized from RNA samples utilizing PrimeScript™ RT reagent Kit (Perfect Real Time) (TaKaRa Bio Inc., Shiga Prefecture, Japan) with random 6-mers and Oligo dT primers, according to the manufacturer’s protocol. Master mix containing forward and reverse primers, SYBR Green I Master Mix, RNAse-free water was prepared on ice and 1 μl of cDNA template (5 × dilution of stock) added individually in a 384-well plate. QRT-PCR amplifications were carried out with QuantStudio 7 Flex Real-Time PCR System (Applied Biosystems, Foster City, CA, United States) with the following cycling conditions: 50°C for 2 min, 95°C for 10 min, 95°C for 15 s with 40 repeats and 60°C for 1 min. All reactions were performed with 3 biological and 2 technical repeats. Primers used are *hif1aa*_For: GCCACACTTTAGACATGCGC; *hif1aa*_Rev: GGCTGTGGT GTGTTTTGGTC; *ca9*_For: ATCCAGAAGACAGCTCGCAG; *ca9*_Rev: TCCCATGCAAAAGTTGTGGTG; *slca1a*_For: TCGA AGCTGGCTGAATCCTG; *slca1a*_Rev: TCGGGGCAATTTCTC CAACA; *vhl*_For: TAGCTCAGAGACCCAGCATT; *vhl*_Rev: CAGTACATATGCACAGTCCAG; *opn1lw2*_For: TGATGGC TCTGAGGT; *opn1lw2*_Rev: TCCAGTTCTTCCCTCTTGTTC; *gnat2*_For: CGTGATCTGAGGTACAGGGC; *gnat2*_Rev: GCT ACCCATCTCGTCGTCTG; *rx2*_For: TCCAGCCCACCTATAC TGCT; *rx2*_Rev: ACTGGTTGGCATTGGTAGGG; β*-actin*_For: CTTCCTGGGTATGGAATCTTGC and β*-actin*_Rev: GTGGAA GGAGCAAGAGAGGTG.

### Mass Spectrometry Analysis

#### Sample Preparation and Mass Spectrometry

Protein was isolated from the eyes of 0.1% DMSO or 100 μM TubA treated siblings (*atp6v0e1*^+^*^/^*^+^ and *atp6v0e1*^+/−^ larvae) and *atp6v0e1*^–^*^/^*^–^ larvae at 6 dpf, using iST Sample Preparation Kit (mammalian tissue, PREOMICS, Martinsried, Germany) according to manufacturer’s protocol. Eyes were dissected from 24 to 30 larvae for protein isolation from each treatment group (*N* = 3). Samples were analyzed by the Mass Spectrometry Resource (MSR) in University College Dublin on a Thermo Scientific Q Exactive mass spectrometer connected to a Dionex Ultimate 3000 (RSLCnano) chromatography system. Peptides were separated on C18 home-made column (C18RP Reposil-Pur, 100 × 0.075 mm × 3 μm) over 150 min at a flow rate of 250 nL/min with a linear gradient of increasing acetonitrile from 1% to 27%. The mass spectrometer was operated in data dependent mode; a high resolution (70,000) MS scan (300–1600 m/z) was performed to select the twelve most intense ions and fragmented using high energy C-trap dissociation for MS/MS analysis.

#### Data Processing and Bioinformatics

Raw data from the Q-Exactive was processed using MaxQuant (Cox and Mann, 2008; Tyanova et al., 2016a) (version 1.6.4.0) incorporating the Andromeda search engine (Cox et al., 2011). To identify peptides and proteins, MS/MS spectra were matched against Uniprot Danio rerio database (2018_05) containing 46,933 entries. All searches were performed using the default setting of MaxQuant, with trypsin as specified enzyme allowing two missed cleavages and a false discovery rate of 1% on the peptide and protein level. The database searches were performed with carbamidomethyl (C) as fixed modification and acetylation (protein N terminus) and oxidation (M) as variable modifications. For the generation of label free quantitative (LFQ) ion intensities for protein profiles, signals of corresponding peptides in different nano-HPLC MS/MS runs were matched by MaxQuant in a maximum time window of 1 min (MAXLFQ) (Cox et al., 2014). The Perseus (Tyanova et al., 2016b) computational platform (version 1.6.2.3) was used to process MaxQuant results. Data was log transformed. To examine changes in protein expression using volcano plots, data was log transformed and missing values were imputed with values from a normal distribution. Data was normalized using *z*-score and visualized using heat maps. In order to evaluate pathway annotation networks of enriched proteins (*t*-test *p*-value < 0.5) from *atp6v0e1*^–^*^/^*^–^ + 100 μM TubA compared with *atp6v0e1*^–^*^/^*^–^ + 0.1% DMSO or *atp6v0e1*^–^*^/^*^–^ + 0.1% DMSO compared with Sibling + 0.1% DMSO, pathway enrichment analysis was performed using the ClueGo (Bindea et al., 2009) (v2.5.2) and Cluepedia (Bindea et al., 2013) (v1.5.2) plugins in Cytoscape (Shannon et al., 2003) (v3.6.1) with the danio rerio (7955) marker set. The KEGG (Ogata et al., 1999) functional pathway databases, consisting of 6,004 genes, were used. GO tree levels (min = 3; max = 8) and GO term restriction (min#genes = 3, min% = 1%) were set and terms were grouped using a Kappa Score Threshold of 0.4. The classification was performed by the right sided-hypergeometric enrichment test, and its probability value was corrected by the Benjamini-Hochberg (Adjusted% Term *p*-value *leq* 0.05).

### Cell Work on Hypoxia

HeLa HRE-GFP^ODD^ cells were maintained in DMEM (Sigma-Aldrich) supplemented with 10% fetal calf serum (Sigma-Aldrich) and 100 units/ml penicillin and 100 μg/ml streptomycin in a 5% CO_2_ incubator at 37°C. HeLa HRE-GFP^ODD^ cells were produced as described previously to facilitate quantitative analysis of Hif-1α activity (Burr et al., 2016). CRISPR sgRNAs were cloned into lentiviral expression vector pKLV-U6sgRNA(*Bbs*I)-pGKPuro2ABFP. Cas9 is stably expressed in the cells using a lentiviral vector (Lenti-CAS9-T2A-Blastocydin) as described previously (Miles et al., 2017). All sgRNA sequences used are ATP6V0E1_guide 1: GCGGCGACCATGGCGTATCA, ATP6V0E1_guide 2: AGTGAGGCCGTGATACGCCA, ATP6V0E1_guide 3: TCACAATGAGAGGCACAGTG and PHD2_guide 1: ATGCCGTGCTTGTTCATGCA. The ATP6V0E1 guides were selected from the Sabatini library and the specificity and efficiency of all guides were predicted using the CRISPOR Tool (Table 1) (Park et al., 2017; Concordet and Haeussler, 2018). HEK293T cells were transfected using TransIT-293 (Mirus Bio, Madison, WI, United States) following manufacturer’s protocol. 2 μg DNA [3:2:4 ratio of the relevant lentiviral transgene vector, pCMVR8.91 (gag/pol) and pMD.G (VSVG)] was used in 6 well plates with cells at 80% confluency in 2 ml media. Viral supernatant was harvested after 48 h, filtered through a 0.45 μM filter and stored at −80°C. Lentiviral transduction was performed by adding 500 μl of virus to 2 × 10^5^ cells in a 24 well plate made up to 1 ml media. Cell plates were centrifuged for 1 h at 37°C at 1,800 rpm immediately after addition of virus. Transduced cells were selected by puromycin treatment. 1 × 10^6^ cells per sample were harvested, washed in 3 ml ice-cold PBS, fixed in 500 μl PBS with 1% paraformaldehyde and analyzed using BD Fortessa cell analyzer for GFP (AF 488, Franklin Lakes, NJ, United States). Samples were labeled with HIF-1α (BD Biosciences, #610959), PHD2 (Novus Biologicals, #NB100-137, Littleton, CO, United States) and β actin (Sigma-Aldrich, #A228) antibodies. Treatment of cells with Fe (III) Citrate and immunoblot assays follows as described previously (Miles et al., 2017). Experiments were carried out twice each.

**TABLE 1:** Bioinformatic analysis of selected sgRNA guides for specificity and off-targets.

		Specificity Score		Off-target counts*	Predicted efficiency
Oligos	Target Sequence	MIT	CFD		Doench ’16-Score
ATP6V0E1 gRNA1	GCGGCGACCATGGCGTATCACGG	93	98	0-0-1-1-9	51
ATP6V0E1 gRNA2	AGTGAGGCCGTGATACGCCATGG	98	96	0-0-0-0-42	62
ATP6V0E1 gRNA3	TCACAATGAGAGGCACAGTGAGG	58	76	0-0-4-28-232	74
PHD2 gRNA	ATGCCGTGCTTGTTCATGCACGG	83	93	0-0-0-4-83	62

*An off-target count of “0-0-1-1-9” means that the target matches 0 locations in the genome with 0 or 1 mismatch. There is 1 location with 2 or 3 mismatches each and 9 regions with 4 mismatches.

### Statistical Analysis

All experiments were performed in triplicate, unless otherwise stated. OKR data was analyzed using either One-way ANOVA with Dunnett’s multiple comparisons or Student’s Unpaired *T*-test in GraphPad Prism 7/8 (La Jolla, CA, United States). QRT-PCR data was analyzed using One-way ANOVA with Dunnett’s multiple comparisons (GraphPad Prism 7) with *p* ≤ 0.05 considered statistically significant. Rodent explant study values in all graphs represent the mean ± standard error of the mean (SEM). Data were statistically analyzed using One-way ANOVA with Dunnett’s multiple comparisons (GraphPad Prism 8) with values of *p* ≤ 0.05 being considered statistically significant.

## Results

### Selective HDAC6 Inhibitors Significantly Restore Cone Photoreceptor Vision in *atp6v0e1^–/–^* Zebrafish Model of Retinal Blindness

**FIGURE 1:**
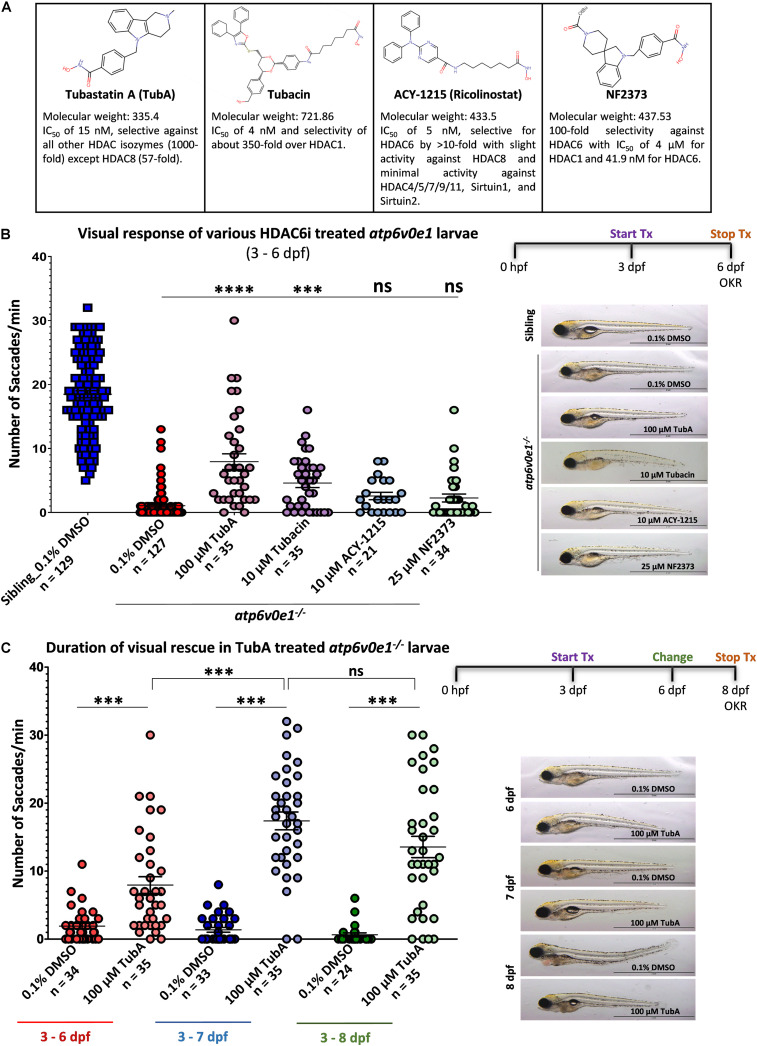
Selective HDAC6i significantly restores visual function in *atp6v0e1^–/–^*, a zebrafish model of retinal blindness. **(A)** Table describing the physiochemical properties of the four HDAC6i candidates selected for present study. Chemical structures were drawn using Chemspider (http://www.chemspider. com/StructureSearch.aspx). **(B)** *atp6v0e1^–/–^* mutants were treated at maximum tolerated concentration (refer to Supplementary Figures S1, S2A) of each HDAC6i, from 3–6 days post fertilization (dpf). Visual function was markedly improved upon treatment with 100 μM TubA, 10 μM Tubacin, 10 μM ACY-1215 or 25 μM NF2373 compared to vehicle-control (0.1% DMSO) treated larvae. Panel on right shows representative larval wholemount images. Statistical analysis was performed using One-way ANOVA with Dunnett’s multiple comparisons, where ^∗∗∗^ *p*-value = 0.0008 and ^****^ means a *p*-value of ≤ 0.0001. *N* = 3 and *n* = 12 per treatment group. **(C)** The duration of action of TubA was determined, whereby *atp6v0e1^–/–^* larvae were treated from 3 to 8 dpf, and visual function measured at 6, 7 or 8 dpf (refer to Supplementary Figure S2B for visual response in siblings). A significant increase in visual response was recorded at 7 and 8 dpf, respectively. Representative larval images presented in the right panel. Student’s *T* test was used for statistical analysis, where ^∗∗∗^ means a *p*-value of ≤ 0.0001. *N* = 3 and *n* = 12 per treatment group.

The ability of small molecule HDAC6 inhibitors (TubA, tubacin, ACY-1215 and NF2373) to restore cone photoreceptor vision was evaluated in *atp6v0e1^–/–^* zebrafish larvae, a genetic model of blindness potentially relevant to IRD and AMD (Figure 1A). The maximum tolerated concentration was determined for each HDAC6i, based on the highest concentration exhibiting no adverse effects on gross morphology of ≥ 80% surviving larvae and the average optokinetic response at 5 days post fertilization (dpf) not reduced by more than 15% (Supplementary Figure S1). *atp6v0e1^–/–^* larvae were treated with these maximum tolerated concentrations from 3–6 dpf. All HDAC6i tested, improved *atp6v0e1^–/–^* visual function. In hierarchical order, visual behavior improved significantly with 100 μM TubA and 10 μM Tubacin treatment producing 7.9 and 4.6 saccades/minute (saccades/min) on average, respectively, compared to 1.1 saccades/min with vehicle control treated *atp6v0e1^–/–^* larvae (Figure 1B; Supplementary Figure S2A). A modest, non-significant increase following 10 μM ACY-1215 (2.6 saccades/min on average) or 25 μM NF2373 (2.3 saccades/min on average) treatment was observed. Treatment with TubA resulted in the highest magnitude rescue of visual function with an average 8-fold increase in saccades. Thereafter, TubA was utilized as a prototypical HDAC6i for subsequent studies. The duration of *atp6v0e1^–/–^* rescue was determined to understand the time frame of sustained neuroprotection. *atp6v0e1^–/–^* larvae were treated from 3 dpf with changes to the drug solution once every 3 days. The studies were stopped at 8 dpf as feeding could not be sustained while on experiment. A progressive increase in visual function was observed in 100 μM TubA-treated *atp6v0e1^–/–^* culminating in an average of 7.9 and 17.4 saccades/min at 6 and 7 dpf, respectively (Figure 1C). Compared to 7 dpf, a non-significant reduction to 13.5 saccades/min was observed at 8 dpf in 100 μM TubA-treated *atp6v0e1* mutants (Figure 1C). There was no significant difference in the average number of saccades between vehicle-control or TubA-treated siblings at 6, 7 or 8 dpf (Supplementary Figure S2B). A significant difference (*p*-value ≤ 0.0001) in the average number of saccades was observed at 6 dpf between 100 μM TubA-treated siblings (17.3 saccades/min) and *atp6v0e1* mutants (7.9 saccades/min) demonstrating that TubA significantly improved vision of *atp6v0e1* mutants, but not to wildtype levels (Figure 1C and Supplementary Figure S2B). Interestingly, at 7 and 8 dpf, there was no significant difference in the average number of saccades between TubA-treated *atp6v0e1^–/–^* and siblings (16.5 saccades/min and 16.3 saccades/min at 7 and 8 dpf, respectively), highlighting the vast improvement in visual capacity in TubA-treated *atp6v0e1* mutants at these time points (Figure 1C and Supplementary Figure S2B). Following TubA treatment, approximately 26% of TubA-treated larvae inflated their swim bladders compared to 100% uninflated swim bladders in vehicle-control *atp6v0e1^–/–^* larvae; and 100 μM TubA-treated *atp6v0e1^–/–^* larvae presented with increased mobility as shown previously (Leyk et al., 2017). All follow-up studies were performed with TubA as this HDAC6i treatment elicited the most efficacious increase in the average number of saccades/min.

### HDAC6 Inhibitor Tubastatin A Improves Retinal Histology and Photoreceptor Ultrastructure

**FIGURE 2:**
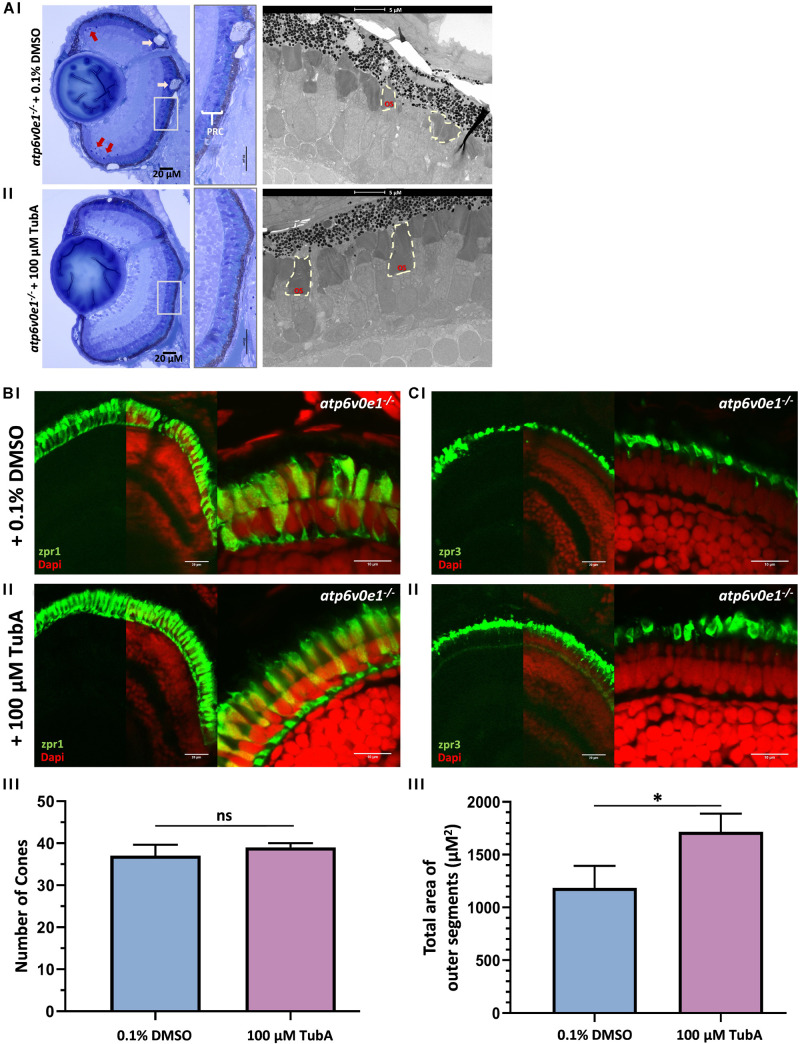
TubA treatment improved retinal morphology in *atp6v0e1^–/–^* larvae. **(Ai)** LM images revealed large vacuoles (pink arrows) in the RPE layer, pyknotic nuclei (red arrows) in the CMZ and shortened photoreceptor outer segments in vehicle-control treated *atp6v0e1*^–/–^ mutants. **(Aii)** The photoreceptor outer segments were observed to be more elongated, more prominent, and better organized in TubA-treated *atp6v0e1*^–/–^ mutants compared to vehicle-control treated *atp6v0e1^–/–^* mutants (Supplementary Figure S3). **(B,C)** Confocal images of immuno-stained vehicle-control or TubA-treated *atp6v0e1^–/–^* mutant retina with cone (zpr-1) photoreceptor and rod and cone outer segment (zpr-3) specific markers. **(Bi,Ci)** Panels present aberrant cone and rod outer segment morphology in vehicle-control treated *atp6v0e1^–/–^* mutants. **(Bii,Cii)** TubA treatment improved photoreceptor structural integrity and organization in *atp6v0e1^–/–^* mutants. **(Biii,Ciii)** Graphical presentation of the number of cone cells (zpr-1 staining) and total area occupied by photoreceptor outer segments (zpr-3 staining), respectively (refer to Supplementary Figure S4 for analysis of retinal histology in siblings; 3 sections per sample was analyzed, *N* = 3). Student’s unpaired *T*-test was used for statistical analysis.

A significant improvement in vision suggested that TubA treatment elicits neuroprotective effects on the retina. Thus, retinal morphology and ultrastructure was analyzed by light and electron microscopy. Vehicle-control treated *atp6v0e1^–/–^* at 6 dpf, had large vacuoles (pink arrows) within the retinal pigment epithelium (RPE) layer, pyknotic nuclei (red arrows) in the ciliary marginal zone (CMZ) and aberrantly formed photoreceptor outer segments (Figure 2Ai). In contrast, we observed that TubA-treated *atp6v0e1^–/–^* displayed improved organization of the photoreceptor layer, with more elongated outer segments and decreased CMZ pyknotic nuclei (Figure 2Aii), validating previous findings (Leyk et al., 2017). TubA improved the arrangement and packing of long double cones and rods in the photoreceptor layer (Figures 2B,C; Supplementary Figure S3). There was no significant increase in the number of photoreceptors following TubA treatment (Figure 2B). The total area occupied by photoreceptor outer segments of cones and rods in TubA-treated *atp6v0e1^–/–^* was significantly larger (*p*-value = 0.027) compared to *atp6v0e1^–/–^* vehicle controls but not restored to sibling levels (Figure 2C; Supplementary Figure 4). In summary, the selective HDAC6i, TubA, improved retinal morphology and photoreceptor ultrastructure consistent with improved visual behavior.

### HDAC6i Tubastatin A Increases Cone Photoreceptor Survival in *rd10* Mouse Retinal Explants

**FIGURE 3:**
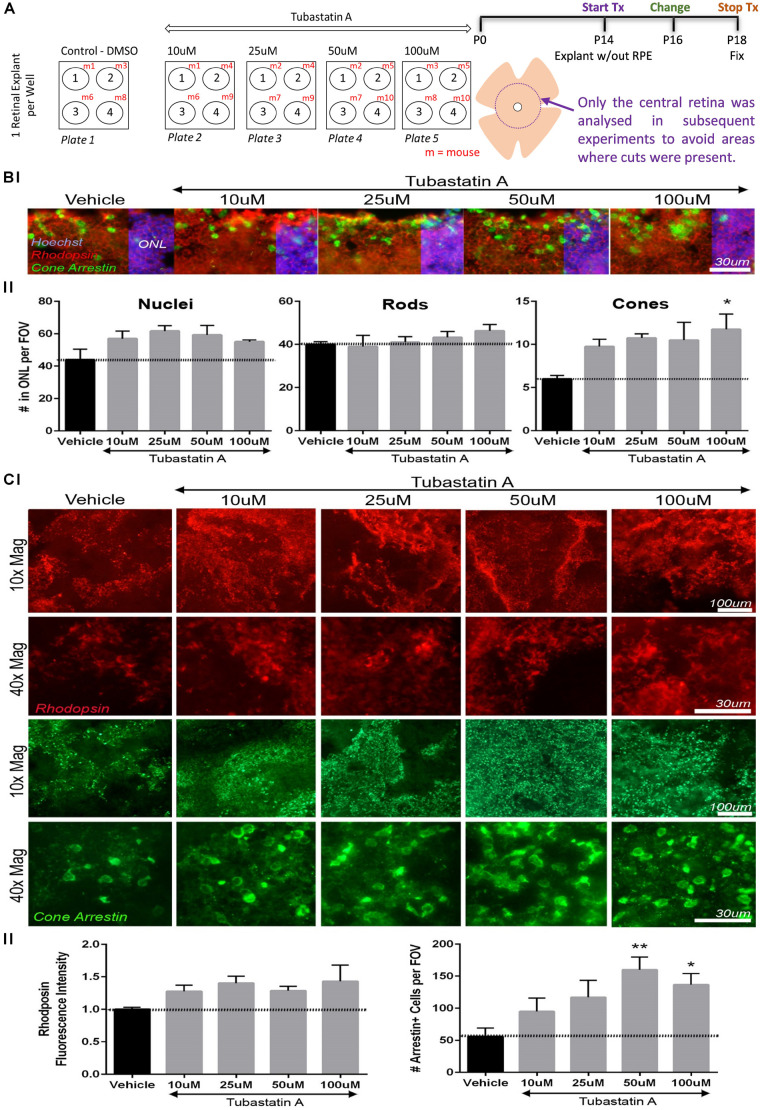
TubA preserves cone cells in *rd10* mice retinal explants. **(A)** Schematic diagram outlining the drug treatment protocol in retinal explants from homozygous *rd10/rd10* mice (B6.CXBI–Pde6brd10/J) at postnatal day 14 (P14). **(B)** Retinal section fluorescent images of explants treated with increasing concentrations of TubA or vehicle control, labeled for rhodopsin (rods) and cone arrestin (cones). The total number of nuclei in the outer nuclear layer (ONL) did not change significantly across the TubA treatment group. Likewise, the number of rhodopsin positive outer segments did not significantly change. A dose-dependent increase in the number of cone arrestin positive outer segments was observed, with a significant increase following 100 μM TubA treatment. **(C)** In wholemount preparations, no significant increase in fluorescence intensity of rhodopsin positive cells, was observed following TubA treatment. A significant increase in the number of cells with cone arrestin positive outer segments was identified at 50 μM and 100 μM TubA treatment. Statistical analysis was performed using One-way ANOVA with Dunnett’s multiple comparisons. *N* = 4 per treatment group.

The capability of TubA to protect against photoreceptor cell death was determined in an *ex vivo* mammalian model of early-stage retinal degeneration, the *rd10* mouse at P14. Retinal explants were prepared from homozygous *rd10/rd10* mice (B6.CXBI−Pde6brd10/J) at postnatal day 14 (P14) as described previously (Roche et al., 2017, 2018; Ruiz Lopez et al., 2017). At this stage *in vivo*, retinal cell types are differentiated and outer segments elaborated in the murine retina (Bassett and Wallace, 2012). Compared to wildtype retina, a significant reduction in the length of rod and cone outer segments occurs in *rd10* retina between P10 and P15, without a significant reduction in ONL thickness (Roche et al., 2016). Hence, P14-P18 was chosen for *rd10* explant culture to assess if TubA improved rod and cone outer segment morphology. Explants were cultured without the RPE in media containing various concentrations of TubA or vehicle control (DMSO), with media/drug change following 2 days in culture and treatment stopped on day 4 of culture (Figure 3A). The RPE supports maturation of early post-natal retinal explant cultures but is not necessary in our experimental paradigm as P14 mouse retinae have already constituted photoreceptor outer segments before culture (Pinzon-Duarte et al., 2000). Explants were fixed and stained with cone and rod markers in retinal wholemounts or sections. The average total number of nuclei in the outer nuclear layer (ONL) ranged from 44 per field of view (FOV) in vehicle control-treated explants to 57, 62, 59 and 55 nuclei per FOV in 10, 25, 50 and 100 μM TubA-treated samples, respectively. The observed changes were not significant (*p*-value = 0.162) (Figure 3B). The average number of cells with rhodopsin-positive outer segments changed from 40 in vehicle control to 39, 41, 43 and 46 cells in 10, 25, 50 and 100 μM TubA-treated explants, but were also not significantly (*p*-value = 0.605) increased (Figure 3B). Interestingly, 100 μM TubA treatment significantly (adjusted *p*-value = 0.024) increased, from an average of 6 in vehicle-treated controls to 12, the number of cone outer segments labeled with cone arrestin (Figure 3B). Wholemount analysis of the explants support the retinal section data, demonstrating a significant (adjusted *p*-value = 0.006 and 0.030, respectively) increase (from 56 to 160 and 137, respectively) in the average number of cells expressing cone arrestin in 50 and 100 μM TubA treatment groups (Figure 3C). On the other hand, no significant (*p*-value = 0.217) difference in the rhodopsin fluorescence intensity was observed between vehicle-control and TubA-treated explants.

### *ATP6V0E1* Subunit Regulates HIF-1α Levels Through Iron

**FIGURE 4:**
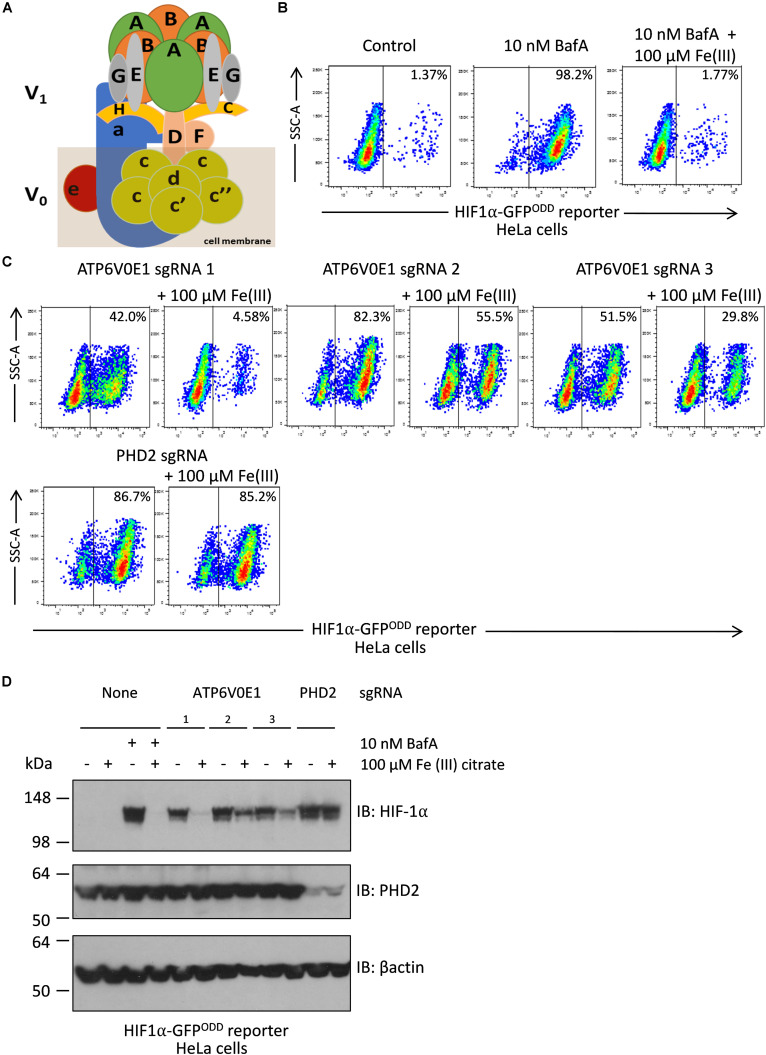
Iron supplementation restores HIF-1α levels to normal following *ATP6V0E1* inhibition in HeLa cells. **(A)** Schematic diagram of the multimeric V-ATPase complex. **(B)** Chemical inhibition of V-ATPase by 10 nM BafA treatment for 24 h, increased HIF-1α levels in HIFα-GFP^ODD^ reporter cells. Treatment with 100 μM Fe (III) citrate significantly reduced the elevated HIF-1α levels associated with loss of *ATP6V0E1* (1 × 10^6^ cells per sample harvested and analyzed; *N* = 2). **(C)** Knock-down of *ATP6V0E1* subunit with three different CRISPR-Cas9 guides resulted in significant upregulation of HIF-1α levels in HIFα-GFP^ODD^ reporter cells. Co-treating cells with 100 μM Fe (III) citrate led to a reduction in HIF-1α levels across the three *ATP6V0E1* depleted cells. *phd2* was knocked down as a control and treatment with 100 μM Fe (III) citrate did not result in reduction of HIF-1α levels (1 × 10^6^ cells per sample harvested and analyzed; *N* = 2). FACs plot shown is a representative image of two biological repeats performed. **(D)** Immunoblot analysis for HIF-1α and PHD2 levels in HIFα-GFP^ODD^ reporter cells with either *ATP6V0E1* or *PHD2* depleted or treated with 10 nM BafA. The cells were treated with 100 μM Fe (III) citrate for 24 h. β actin was used as a control. Results validated findings observed by flow cytometry, whereby HIF-1α levels were upregulated following *ATP6V0E1* knock-down or inhibition and levels were re-normalized upon Fe (III) citrate treatment. Treatment of Fe (III) citrate in *PHD2* depleted cells did not alter HIF-1α levels. All experiments were performed in biological duplicate.

Recently, V-ATPase inhibition or depletion of other subunits of the multi-functional complex were reported as regulators of HIF-1α levels in a process related to levels of free intracellular iron (Miles et al., 2017). To quantitatively determine if the human ATP6V0E1 subunit ortholog regulated HIF-1α activity, comparable to other V-ATPase subunits, we used the dynamic HIF-1α fluorescent reporter (HeLa HRE-GFP^ODD^ cells) cell line, as described previously (Burr et al., 2016). The HeLa HRE-GFP^ODD^ cell line was selected for this experiment as the cell line has validated, bespoke modifications incorporating a HIF-1α driven reporter transgene allowing for quantitative analysis of HIF-1α activity and direct comparison of the role of the ATP6V0E1 subunit with other V-ATPase subunits in the same cell line. As expected, pharmacological inhibition of the V-ATPase complex with 10 nM bafilomycin A1 (BafA) led to a significant increase in HIF1α-GFP^ODD^ reporter activity in HeLa cells, 98.2% HIF1α-GFP positive cells compared to 1.4% in untreated controls (Figures 4A,B). Co-supplementing cells treated with 10 nM BafA and 100 μM of Fe (III) citrate, significantly reduced HIF1α-GFP positive cells to 1.77%, consistent with HIF-1α stability being dependent on intracellular iron abundance. We then investigated if the human *ATP6V0E1* ortholog regulates HIF-1α levels. Lentiviral knock-down of *ATP6V0E1* with three CRISPR-Cas9 guides was performed resulting in increased HIF-1α activity (42.0%, 82.3% and 51.5%, respectively, with *atp6v0e1* guides 1, 2 and 3) as measured by HIF1α-GFP^ODD^ reporter activity in HeLa cells (Figure 4C). This effect was reversed (HIF-1α levels were reduced to 4.58, 55.5, and 29.8%, respectively) by co-treatment with 100 μM Fe (III) citrate (Figure 4C). *PHD2* depleted cells were used as a negative control, whereby knock-down of *PHD2* resulted in a significantly higher number of HIF1α-GFP positive (86.7%) cells and, as expected, treatment with 100 μM Fe (III) citrate did not reduce the number of HIF1α-GFP positive (85.2%) cells (Figure 4C). Immunoblotting confirmed that Fe (III) citrate treatment reduces HIF-1α levels in BafA treated cells and *ATP6V0E1* depleted cells (Figure 4D). However, Fe (III) citrate does not alter HIF-1α levels in *PHD2* depleted cells. This data revealed that the *ATP6V0E1* subunit functionally regulates HIF-1α levels *in vitro*.

### HDAC6 Inhibitor Tubastatin A Does Not Modulate *hif-1α* Target Transcripts in *atp6v0e1^–/–^* Eyes at 3 or 6 dpf

**FIGURE 5:**
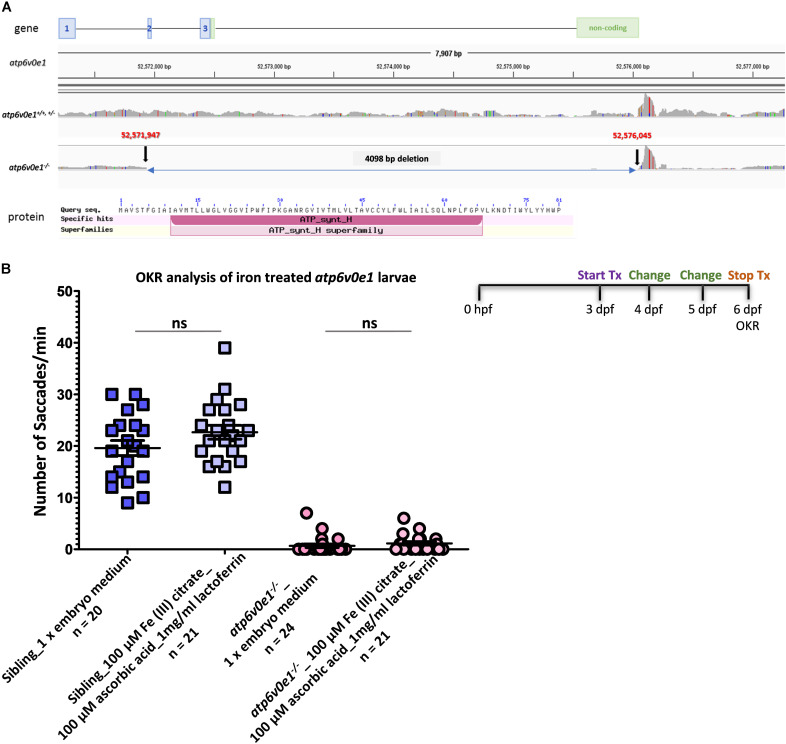
Iron supplementation did not restore vision in *atp6v0e1* mutants. **(A)** Schematic diagram depicting the 4,098 bp deletion in the *atp6v0e1* subunit identified through whole genome sequencing. **(B)** Treatment of *atp6v0e1^–/–^* with 100 μM Fe (III) citrate, co-supplemented with 100 μM ascorbic acid and 1 mg/ml of lactoferrin daily for 3 days, did not restore visual function as measured by OKR. Experiments were performed in duplicate and Student’s *T*-Test was used for statistical analysis.

Whole genome sequencing was performed to define the genetic deletion in *atp6v0e1*^–^***^/^****^–^* larvae. Bioinformatic analysis identified an approximately 4,098 base pair deletion, spanning from exon 2 to the 3′ UTR region of *atp6v0e1* (Figure 5A). Previously, we reported *atp6v0e1^–/–^* results from a 180 base pair deletion, but this was due to a spurious transcript arising from PCR amplification (Daly et al., 2017). The large deletion identified by genome sequencing is predicted to introduce a premature stop codon and induce nonsense mediated transcript decay. *atp6v0e1* transcripts are significantly reduced in *atp6v0e1^–/–^* compared to siblings (unpublished data).

We hypothesized that lack of a functional *atp6v0e1* will result in elevated Hif-1α levels, *in vivo*. To link with our earlier finding that Fe (III) citrate normalizes HIF-1α reporter activity, upregulated *in vitro* by *ATP6V0E1* depletion, we co-treated *atp6v0e1****^–/–^*** zebrafish with 100 μM Fe (III) citrate, 100 μM ascorbic acid and 1 mg/ml of lactoferrin from 3–6 dpf, replacing drug solutions daily. Supplementing iron failed to significantly rescue *atp6v0e1*^–^***^/^****^–^* visual function at 6 dpf (Figure 5B). Average saccades/min of 0.7 or 1.1 saccades/min were obtained in vehicle-control or iron supplemented *atp6v0e1*^–^***^/^****^–^* larvae, respectively.

**FIGURE 6:**
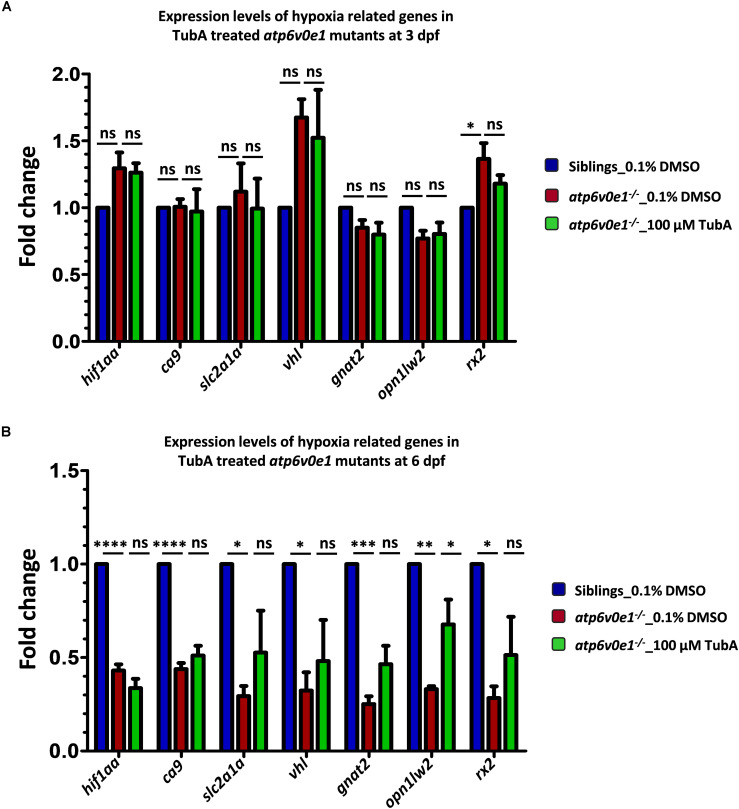
Analysis of *hif1aa* and targets expression levels in *atp6v0e1^–/–^* treated with TubA. **(A)** *hif1aa* and select *hif1aa* downstream target gene(s) expression levels were quantified by qRT-PCR at 4 hpt (*i.e.*, at 3 dpf). No significant changes in *hif1aa*, *slc2a1a*, *vhl* or *ca9* transcript levels occurred in *atp6v0e1^–/–^* in comparison to vehicle-control treated siblings. Treatment with TubA did not significantly alter these transcript levels either. In *atp6v0e1^–/–^*, retinal-specific genes *gnat2* and *opn1lw2* were not significantly changed but *rx2* was significantly upregulated. Upon treatment with TubA, no significant reduction in *rx2* transcript levels was detected. **(B)** Similarly, *hif1aa* and select *hif1aa* downstream target gene(s) expression levels were quantified at 3 dpt (*i.e.*, at 6 dpf). A significant reduction in *hif1aa, ca9*, *slc2a1a* and *vhl* transcript levels was identified in vehicle-control treated *atp6v0e1^–/–^*. Retinal-specific genes *gnat2*, *opn1lw2* and *rx2* were also significantly downregulated compared to vehicle-control treated siblings. TubA-treatment of *atp6v0e1^–/–^* resulted in no significant change in *hif1aa*, *ca9*, *slc2a1a* and *vhl* levels. *opn1lw2* transcript levels were significantly increased following TubA-treatment. All experiments were performed in triplicates (*n* = 60 eyes/treatment condition) and one-way ANOVA with Dunnett’s multiple comparisons was used for statistical analysis.

To test the related theory that an upregulation of HIF-1α arises in *atp6v0e1*^–^***^/^****^–^* zebrafish eyes and contributes to retinal blindness, the transcript levels of *hif1aa* and *hif1aa* target genes (*ca9*, *slc2a1a* and *vhl*) were quantified by qRT-PCR at two time-points. Treatment started at 3 dpf and RNA was extracted from vehicle-control or TubA-treated eyes at 4 h post treatment (hpt) or 3 days post-treatment (dpt). In vehicle-control treated *atp6v0e1*^–^***^/^****^–^* larvae compared to siblings, there was no significant transcript increase in *hif1aa* or *hif1aa* target genes (*ca9*, *slc2a1a* and *vhl)* at 4 hpt (Figure 6A). But *hif1aa* (2.3 fold; adjusted *p*-value ≤ 0.0001) and the *hif1aa* target genes (*ca9*, *slc2a1a* and *vhl;* 2.3, 3.4 and 3.1 fold, respectively; adjusted *p*-value ≤ 0.0001, 0.0168 and 0.0241, respectively) were significantly downregulated at 3 dpt (*i.e.*, 6 dpf) (Figure 6B). We then investigated if HDAC6i treatment alters HIF-1α signaling. However, treatment with 100 μM TubA did not significantly alter *hif1aa* or the *hif1aa* target genes (*ca9*, *slc2a1a* and *vhl)* in *atp6v0e1*^–^***^/^****^–^* at 4 hpt or 3 dpt (Figures 6A,B). Three markers of retinal differentiation, *gnat2*, *opn1lw2* and *rx2* were also analyzed. A significant increase (1.4 fold; adjusted *p*-value = 0.0275) in *rx2* transcripts were observed in *atp6v0e1****^–/–^*** larvae compared to vehicle-control treated siblings at 4 hpt (Figure 6A). Treatment with 100 μM TubA did not significantly change *gnat2*, *opn1lw2* and *rx2* levels at 4 hpt. In contrast, all three genes were significantly downregulated (*gnat2*, *opn1lw2* and *rx2*; 4.0, 3.0 and 3.5 fold, respectively; adjusted *p*-value = 0.0003, 0.0016, and 0.0113, respectively) in *atp6v0e1^–/–^* at 3 dpt (Figure 6B). A significant increase (2.0 fold; adjusted *p*-value = 0.0346) in *opn1lw2* but not *gnat2* or *rx2* was observed in *atp6v0e1****^–/–^*** after HDAC6i treatment.

In summary, there is no evidence that loss of *atp6v0e1 in vivo* alters Hif-1α signaling or phototransduction gene expression in 3 dpf eyes, with or without HDAC6i treatment. However, at 6 dpf, zebrafish knock-outs of *atp6v0e1* display significantly reduced levels of transcripts for Hif-1α and phototransduction signaling genes. HDAC6i treatment from 3–6 dpf is sufficient to significantly increase transcript levels of the red-sensitive opsin gene (*opn1lw2*) in *atp6v0e1^–/–^*larvae, consistent with the improved retinal histology and visual behavior.

### Proteome Profiling Revealed That Tubastatin A Modulates Several Cellular Processes to Mediate the Observed Rescue of Vision

**FIGURE 7:**
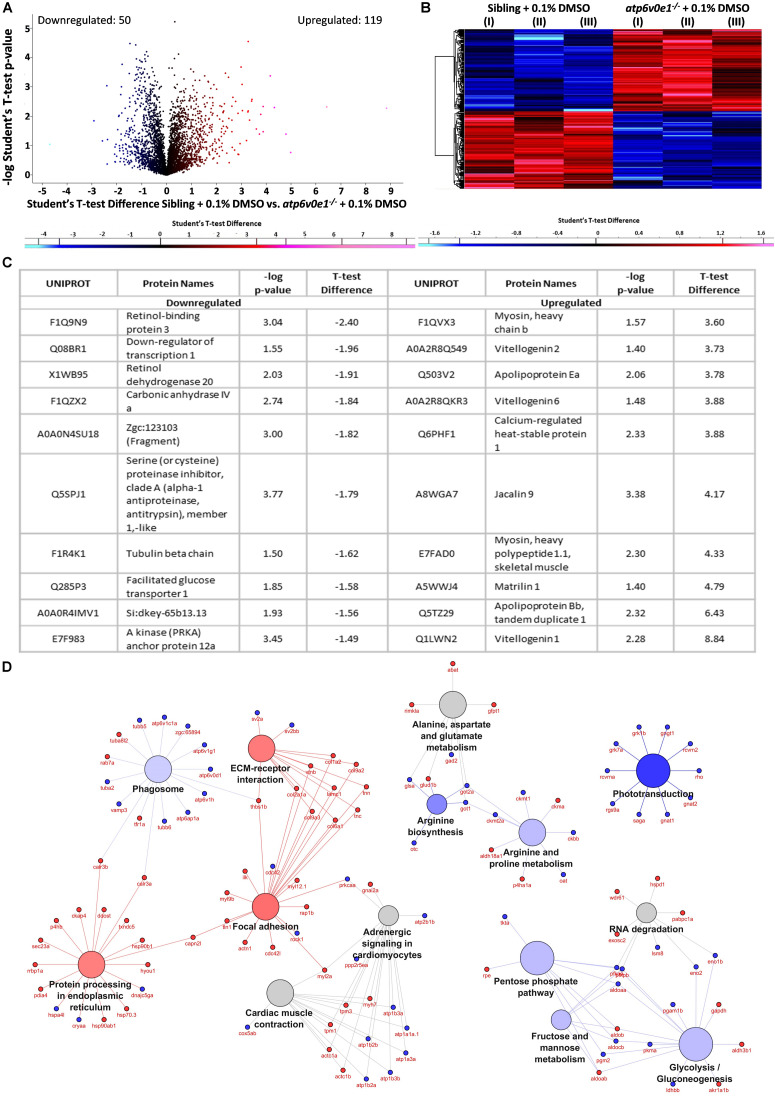
Multiple pathways are implicated in the disease pathomechanism of *atp6v0e1^–/–^* as identified by proteome profiling. **(A,B)** Volcano plot and heatmap representing 790 differentially expressed proteins between vehicle-control treated siblings and *atp6v0e1^–/–^*, out of which, 119 proteins were upregulated (in red) and 50 downregulated (in blue) significantly, given a cut-off of fold change ± 1.2 and *–*log *p*-value ≥ 1.3. Student’s *T*-Test was used for statistical analysis. **(C)** Table describing the top-most significantly downregulated and upregulated proteins. **(D)** KEGG pathway analysis of significantly downregulated and upregulated proteins in vehicle-control treated *atp6v0e1*^–/–^ compared to vehicle-control treated siblings.

As an unbiased approach to understand ocular disease mechanisms associated with *atp6v0e1^–/–^* and therapeutic mechanisms associated with HDAC6 inhibition, proteome profiling was performed. In 6 dpf eyes, a total of 4611 proteins were identified following liquid chromatography mass spectrometry analysis. 790 proteins were statistically altered, out of which 119 ocular proteins were significantly upregulated and 50 downregulated when a cut-off of ± 1.2 fold change and − log *p*-value ≥ 1.3 was applied, between siblings and *atp6v0e1****^–/–^*** knock-outs, as presented by volcano plot and heatmap (Figures 7A,B). The most downregulated ocular proteins in *atp6v0e1^–/–^* include regulators of the vitamin A/retinoid cycle (retinol binding protein 3, retinol dehydrogenase 20) and metabolism (facilitated glucose transporter 1) (Figure 7C). The most upregulated ocular proteins in *atp6v0e1^–/–^* include proteins involved in antioxidant activity, estrogen signaling (vitellogenin 1, 6 and 2), muscle movement (myosin, heavy polypeptide 1.1, skeletal muscle and myosin, heavy chain b) and lipid metabolism (apolipoprotein Bb and apolipoprotein Ea) (Figure 7C). To probe deeper, we analyzed differentially expressed pathways or cellular processes linked with disease pathogenesis in *atp6v0e1^–/–^* larvae. KEGG pathway analysis of all differentially expressed proteins identified proteins involved in phototransduction (*e.g.*, Rho, Gnat1, Gnat2, Gngt1, Grk1b, Rcvrn2, Rgs9a and Saga), phagocytosis (*e.g.*, Atp6ap1a, Atp6v0d1, Atp6v1c1a, Atp6v1g1, Atp6v1h, Aalr3a, Tubb6, Tuba2 and Vamp3), arginine biosynthesis (*e.g.*, Glsa, Got1, Got2a and Otc), arginine and proline metabolism (*e.g.*, Ckbb, Ckmt1, Ckmt2a, Got2a and Oat), glycolysis/gluconeogenesis (*e.g.*, Aldoaa, Eno1b, Eno2, Pgam1b and Pkma), fructose and mannose metabolism (*e.g.*, Aldoaa, Aldocb, Pfkpa, Pfkpb) and pentose phosphate pathway (*e.g.*, Aldocb, Pfkpb, Pgm2 and Tkta), as significantly downregulated in *atp6v0e1^–/–^* (Figure 7D). Protein processing in endoplasmic reticulum (*e.g.*, Calr3b, Ddost, Hsp70.3, Hsp90ab1, Hsp90b1, Hyou1, Rrbp1a and Sec23a), ECM-receptor interaction (*e.g.*, Col2a1a, Col9a3, Lamc1, Thbs1b, Tnc and Vtnb) and focal adhesion (*e.g.*, Actn1, Col2a1a, Ilk, Lamc1, Myl12.1, Thbs1b, Tnc and Vtnb) pathways were upregulated (Figure 7D).

**FIGURE 8:**
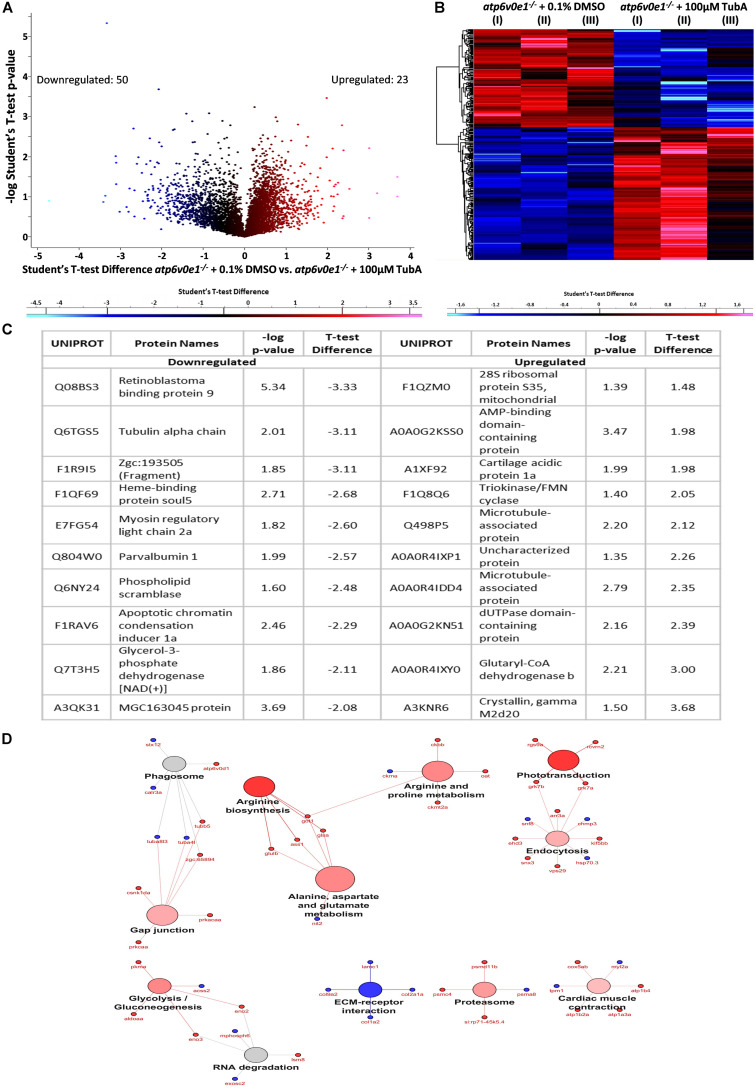
TubA plays a multifaceted role to improve visual function and preserve photoreceptors. **(A,B)** A total of 347 proteins were identified to be differentially expressed between 3–6 dpf vehicle-control and TubA-treated *atp6v0e1^–/–^*. 23 proteins were upregulated (red) and 50 proteins downregulated (blue), with a cut-off of fold change ± 1.2 and –log *p*-value ≥ 1.3, as shown by the volcano plot and heatmap. Student’s *T*-Test was used for statistical analysis. **(C)** Table listing the most significantly down- and upregulated proteins. **(D)** Graphical representation of significantly downregulated and upregulated pathways in TubA-treated *atp6v0e1^–/–^* as identified by KEGG pathway analysis.

Similarly, we analyzed the most differentially expressed proteins in *atp6v0e1*^–/–^ after HDAC6i treatment. Given a cut-off of ± 1.2, and a *−*log *p*-value of ≥ 1.3, 23 and 50 proteins were up or downregulated, respectively, out of 347 proteins that were identified to be statistically changed (Figures 8A,B). The most downregulated ocular proteins in TubA-treated *atp6v0e1^–/–^* include retinoblastoma binding protein 9, tubulin alpha chain and myosin regulatory light chain 2 (Figure 8C). The most upregulated ocular proteins in HDAC6i treated *atp6v0e1^–/–^* include Glutaryl-CoA dehydrogenase, 28S ribosomal protein, microtubule-associated protein(s) and Triokinase/FMN cyclase. Pathway analysis identified the most enriched GO terms as phototransduction (*e.g.*, Grk7a, Grk7b, Rcvrn2 and Rgs9a), cardiac muscle contraction (*e.g.*, Atp1a3a, Atp1b2a, Atp1b4 and Cox5ab), endocytosis (*e.g.*, Arr3a, Ehd3, Grk7a, Grk7b, Kif5bb, Snx3 and Vps29), proteasome (*e.g.*, Psmc4, Psmd11b and Si:rp71-45k5.4) and gap junction (*e.g.*, Csnk1da, Prkacaa, Prkcaa and Zgc:65894). Interestingly, proteins involved in metabolism such as arginine biosynthesis (*e.g.*, Ass1, Glsa, Glulb and Got1), arginine and proline metabolism (*e.g.*, Ckbb, Ckmt2a, Got1, Oat), alanine, aspartate and glutamate metabolism (*e.g.*, Ass1, Glsa, Glulb and Got1) and glycolysis/gluconeogenesis (*e.g.*, Aldoaa, Eno2, Eno3 and Pkma) were significantly upregulated (Figure 8D). ECM-receptor interaction (*e.g.*, Col1a2, Col2a1a, Col9a2 and Lamc1) was downregulated, and phagosome pathway (*e.g.*, Atp6v0d1, Calr3a, Stx12, Tuba4l, and Zgc:65894) was moderately altered (Figure 8D). From the pathway analysis, it was evident that cellular processes such as phototransduction, metabolism and phagocytosis that were dysregulated in the *atp6v0e1^–/–^* eyes, were positively restored and proteasome and endocytosis pathways were increased upon HDAC6i treatment.

## Discussion

Here, we report that selective inhibitors of HDAC6 restore functional cone photoreceptor vision in a zebrafish model of blindness arising from deletion of the *atp6v0e1* subunit of V-ATPase and restore cone photoreceptor survival in a mouse IRD model arising from a missense mutation of the catalytic phosphodiesterase 6B subunit. The finding that HDAC6i can primarily preserve cone function/cells in these diverse animal models is intriguing, given that zebrafish predominantly uses cone mediated color vision while mice have rod-dominant vision (Bibliowicz et al., 2011; Huberman and Niell, 2011). Additionally, the positive effects observed in our *in vivo* zebrafish model and *ex vivo* rodent model are indicative that HDAC6i may be of clinical relevance, an opportunity that needs to be explored.

The beneficial effect of HDAC6 inhibition on cone photoreceptors in the zebrafish *atp6v0e1*^–/^*^–^* and *rd10* mouse models indicates some common mechanisms of disease. One candidate is elevated HIF-1α signaling, a hallmark of IRD and AMD arising from hypoxia within the eye (Lange et al., 2011; Trachsel-Moncho et al., 2018). Reassuringly, elevated HIF-1α protein levels in retinae of *rd10* mice was previously reported (Wang et al., 2014). A more recent publication, reported a cyclical pattern of retinal HIF-1α expression in *rd10* retinae, wherein expression was lower than wildtypes but increased Hif-1α levels correlated with two phases of photoreceptor degeneration (Olivares-Gonzalez et al., 2018). In relation to *atp6v0e1*, genetic knock-down of other V-ATPase subunits resulted in increased Hif-1α levels *in vitro* (Miles et al., 2017). Our research reveals CRISPR-Cas9 knock-down of the *ATP6V0E1* subunit in Hela cells is sufficient to increase Hif-1α levels, an effect mimicked by pharmacological (BafA) inhibition of the V-ATPase complex. However, *in vivo, hif1aa* and its targets (*ca9, slc2a1a* and *vhl*) were not significantly upregulated in *atp6v0e1^–/–^* eyes at 3 dpf and were significantly downregulated at 6 dpf. Overall, our data does not rule out an *in vivo* pathological role for HIF-1α signaling in *atp6v0e1^–/–^;* it appears there is a potential oscillating pattern akin to *rd10* mice and cell-specific Hif-1α alterations are potentially masked by analysis performed on whole eyes. We considered whether HDAC6 inhibition could be mediating beneficial effects by modulating HIF-1 signaling in the retinae. Evidence in support is that HDAC6 promotes HIF-1α transcriptional activity *in vitro*, a unique feature of this Class IIb HDAC, and contrasting with Class I and IIa HDACs which increase HIF-1α stability (Chen and Sang, 2011; Schoepflin et al., 2016). Furthermore, inhibition of HDAC6 in *Vhl^–/–^* human renal carcinoma cells led to reduced HIF-1α expression and activity (Qian et al., 2006). Here, *in vivo*, Tubastatin A treatment did not significantly modulate transcript levels of *hif1aa* or selected downstream targets in *atp6v0e1^–/–^* retinae, albeit cell-specific changes may be masked. Additionally, we show that elevated levels of Hif-1α levels upon knock-down of the *ATP6V0E1* subunit, are regulated by iron supplementation in HeLa cells. *In vivo*, in the *atp6v0e1^–/–^* larvae under our treatment regime, we did not observe an improvement in visual function. However, this could be attributed to multiple caveats involved in our iron treatment regime in zebrafish, *e.g.* inability to quantify iron levels absorbed by the larvae or intracellular iron levels in the retinae to ensure that sufficient iron was delivered to the eyes to be effective. Furthermore, the timepoint at which iron is supplemented and the treatment duration needs to be optimized, given that there could potentially be a modulation of *hif1aa* levels over time. Therefore, we cannot rule out the possible role of iron regulating retinal *hif1aa* levels *in vivo*, and future experiments need to address these limitations.

An alternative common mechanism of disease in zebrafish *atp6v0e1^–/–^* and mouse *rd10* models is altered autophagy, a survival pathway responding to cellular stress (Moreno et al., 2018). The V-ATPase is a key component of autophagy; its proton pumping activity allows activation of lysosomal acid hydrolases which degrade cargo from autophagosomes (Mijaljica et al., 2011). Although PDE6 does not directly regulate autophagy, previous reports document increased levels of autophagy in *rd10* retinae (Rodriguez-Muela et al., 2015; Trachsel-Moncho et al., 2018). We questioned if HDAC6 inhibition could modulate altered processes related to autophagy in *atp6v0e1^–/–^* or *rd10* retinae. In iPSC-derived RPE cells, the pan-HDAC inhibitor valproic acid restored outer segment phagocytosis levels which were blocked by the V-ATPase inhibitor bafilomycin-A1 (Singh et al., 2015). Here, proteomic profiling of *atp6v0e1^–/–^* eyes shows moderately reduced levels of phagosome proteins compared to wildtype siblings, and HDAC6 inhibition resulted in moderately increased levels of phagosome proteins in *atp6v0e1^–/–^* eyes.

Collectively, there is reason to consider HIF-1 signaling and autophagy as therapeutic targets of HDAC6i for *atp6v0e1^–/–^* or *rd10* related blindness, but with potential applicability to a wider cohort of IRD and AMD patients. However, caution must be exercised as there is controversy regarding both pathways. Several studies show attenuating Hif-1α signaling has beneficial effects for photoreceptor-related blindness. Conditional knock-out of *Vhl* in mouse rod photoreceptors, led to increased Hif-1α levels; an anti-*Hif1*α shRNA protected cells from the associated photoreceptor degeneration (Barben et al., 2018a). Moreover, in a mouse model mimicking AMD pathogenesis, inhibiting *Vhl* and *Hif1*α prevented cone photoreceptor degeneration and abnormal vessel growth (Barben et al., 2018b). However, in contrast, others report that stabilizing HIF-1 signaling prevents retinal degeneration. Stabilization of Hif-1α protected against photoreceptor cell death in pre-clinical models of retinal detachment and retinopathy of prematurity (Hoppe et al., 2016; Liu et al., 2016; Cheng et al., 2017). Indeed, in *rd10* mice, pharmacologically inhibiting HIF-1α degradation with dimethyloxalylglycine (DMOG) prevented photoreceptor loss (Olivares-Gonzalez et al., 2018). In relation to autophagy, conditional knock-out of *Atg5* (autophagy gene involved in the formation of autophagosomes) in murine rod photoreceptors resulted in the accumulation of visual transduction proteins and consequent degeneration of photoreceptors (Yao et al., 2016). In *rd10* mice, promoting autophagy with rapamycin, increased retinal cell death (Rodriguez-Muela et al., 2015). However, other studies report autophagy is increased as a mechanism to clear out mutant proteins or damaged cells or as a response to oxidative stress (Kaushal, 2006; Kunchithapautham and Rohrer, 2007; Bogea et al., 2015). Hence, a balance is required in autophagy in diseased conditions to ensure a beneficial effect (Moreno et al., 2018; Lin and Xu, 2019).

In addition to these candidate disease pathways, unbiased proteomic profiling identified phototransduction and metabolic pathways, linked with HDAC6i rescue of *atp6v0e1*^–/^*^–^*. However, these pathways can be viewed from different perspectives, either as a cause or consequence. In vehicle-control treated *atp6v0e1*^–/^*^–^*, proteins in the phototransduction pathway were downregulated, which implies that either there is a lack of phototransduction related proteins (cause) leading to the reduced visual function (consequence) or that the aberrant morphology/degenerating photoreceptors (cause) resulted in the reduced expression of retinal protein leading to vision loss (consequence). Reassuringly, phototransduction-specific proteins were upregulated upon TubA treatment which supports the observed increase in functional vision. This could mean either (1) TubA is neuroprotective of retinal cells (cause), leading to increased protein levels and activity (consequence) or (2) the increase in expression of phototransduction proteins (cause) supports the observed preservation and activity of photoreceptors (consequence) in the retina. We additionally analyzed expression levels of select inner retinal proteins (*i.e.*, Slc6a9, Calb2a, Calb2b, Snap25a, Prkca and Vim) and found that these proteins were not altered in *atp6v0e1* mutants. This further supports the notion that defects in photoreceptors are causative of the compromised visual function in the mutants and our findings need to be further investigated in follow up studies.

Interestingly, TubA increased expression levels of metabolic proteins involved in arginine biosynthesis, alanine, aspartate and glutamate metabolism and glycolysis/gluconeogenesis in *atp6v0e1* mutants. Functional vision is an energy-demanding process and photoreceptors are known to utilize glucose primarily as their energy source (Narayan et al., 2017). The observed increase in metabolic processes may correlate to improved energy consumption of functional retinal cells in *atp6v0e1^–/–^* following HDAC6 inhibition. Several proteins involved in a variety of metabolic pathways were downregulated in vehicle-control treated *atp6v0e1^–/–^*. This could mean either (1) there was an insufficient amount of energy sources (cause) in the retina leading to non/reduced retinal functions (consequence) or (2) the reduction in proteins related to metabolic pathway(s) observed (consequence) is due to the reduced visual function of retinal cells (cause). Likewise, the observed increase in levels of metabolic proteins following treatment with TubA indicates that either (1) TubA increased the levels of proteins responsible for providing energy sources (cause) in the retina leading to increased retinal functions (consequence) or (2) as there is increased retinal function, due to the preservation of retinal cells (cause), there is an increase in energy consumption (consequence) by the retinal cells. As the pathways identified from our study, could be addressed as either the cause or consequence, we are proposing that HDAC6i plays a multifaceted role in ensuring the preservation and function of retinal cells. This highlights the need to identify and validate driver pathways mediating restoration of vision through HDAC6i by profiling changes at earlier time points.

In summary, our data provides evidence that HDAC6i are efficacious as photoreceptor neuroprotectants whose potential as a therapeutic option for retinal and macular degenerations warrants further investigation. Indeed, as loss of cone photoreceptor vision is associated with the most detrimental impact on vision in IRD and AMD, our findings suggest HDAC6 inhibitors and associated target pathways offer potential treatments to patients affected by these blinding conditions. Important questions to address in the future are the safety and efficacy profile of HDAC6i, evaluation of the impact of HDAC6i treatment on long term visual function and intraocular delivery of HDAC6i in rodent models, *in vivo*, to fully appreciate the therapeutic potential of HDAC6i for treatment of IRD and AMD. As HDAC6i are selective and act on non-histone proteins, side effects associated with pan-HDACi are less concerning. With over 270 genes causative of IRD, HDAC6 inhibition offers potential for a wider subset of patients than single gene therapies. However, both the disease mechanisms and the stage of disease must be taken into consideration.

## Data Availability Statement

The raw data supporting the conclusions of this article will be made available by the authors, without undue reservation, to any qualified researcher.

## Ethics Statement

The animal study was reviewed and approved by UCD Animal Research Ethics Committee, University College Dublin, Ireland; UCC Animal Experimentation Ethics Committee, University College Cork, Ireland; and Health Products Regulatory Authority (HPRA), Ireland.

## Author Contributions

HS and BK were responsible for study conceptualization, wrote the original draft, performed the project administration. HS, SR, GC, JN, and BK led funding acquisition. HS, SR, GG, JN, and BK led the methodology. HS, SR, GG, and ED led the formal analysis and visualization. HS, SR, GG, AM, and ED performed the investigation. JN and BK led the acquisition of resources. HS, SR, GG, AM, ED, GC, JN, and BK wrote, reviewed, and edited the manuscript. BK acted as lead for supervision. All authors contributed to manuscript revision, read and approved the submitted version.

## Conflict of Interest

The authors declare that the research was conducted in the absence of any commercial or financial relationships that could be construed as a potential conflict of interest.

## Abbreviations

AD: Alzheimer’s disease
AMD: Age-related macular degenerations
BafA: Bafilomycin A1
CMZ: Ciliary marginal zone
DMSO: Dimethyl sulfoxide
dpf: Days post fertilization
FOV: Field of view
HDAC6i: Histone deacetylase 6 inhibitors
HDACi: Histone deacetylase inhibitors
HDACs: Histone deacetylases
HPT: Hours post treatment
IRD: Inherited retinal degenerations
MTC: Maximum tolerated concentration
OKR: Optokinetic Response
ONL: Outer nuclear layer
PFA: Paraformaldehyde
RP: Retinitis pigmentosa
RPE: Retinal pigment epithelium
RPM: Revolutions per minute
RT: Room temperature
Saccades/min: Saccades/minute
TubA: Tubastatin A
V-ATPases: Vacuolar H^+^ ATPases.
