# Supplementary material for: Selective Histone Deacetylase 6 Inhibitors Restore Cone Photoreceptor Vision or Outer Segment Morphology in Zebrafish and Mouse Models of Retinal Blindness
Source: Front Cell Dev Biol. 2020 Aug 26;8:689. doi: 10.3389/fcell.2020.00689 (PMC7479070; doi:10.3389/fcell.2020.00689)
Supplement: Supplementary file 1 [file Data_Sheet_1.pdf]

Supplementary Figures

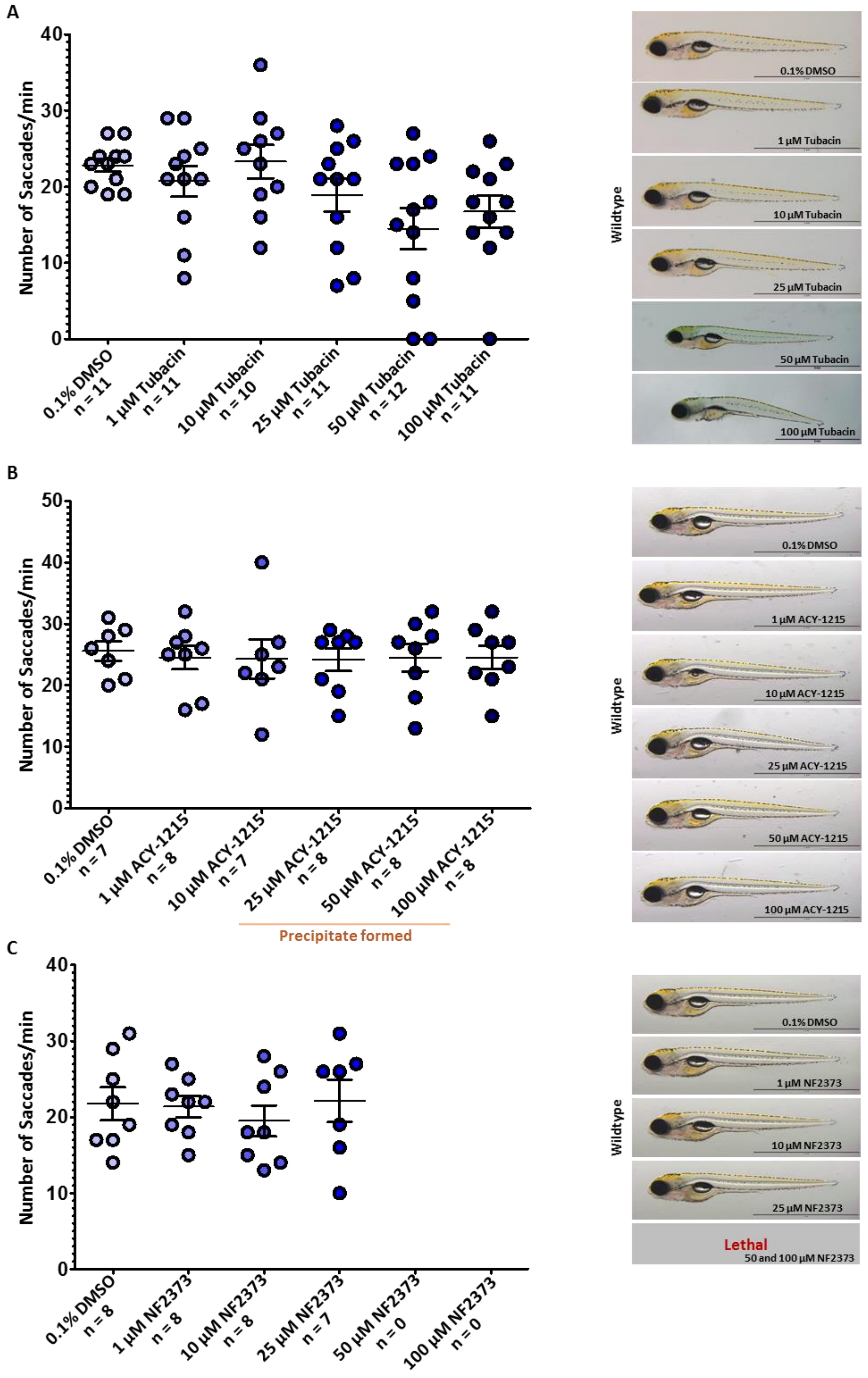

Supplementary Figure 1: Determining maximum tolerated concentration of various HDAC6i compounds in wildtype larvae. (A - C) Wildtype larvae were treated for 3 days

from 3 dpf with selected HDAC6i, at increasing (1, 10, 25, 50 and 100 µM) doses and 0.1% DMSO was used as vehicle control (N = 1, n = 8 - 12 larvae/treatment group). MTC was determined based on the given criteria 1) an adverse effect was not observed in overall morphology of all surviving (> 80% survival rate) larvae and 2) visual function should not reduce by >15% of average number of saccades. (A) In all concentrations of tubacin, the larvae presented with a visual response, 10 µM of Tubacin treatment was selected as the MTC, as concentrations at 25 µM and higher resulted in a reduction in average number of saccades, which could be indicative of potential toxicity in the eye. (B) HDAC6i ACY-1215 precipitated at higher concentrations (25, 50 and 100 µM), though a visual response was obtained at said concentrations and the larvae did not present with toxicity. 10 µM ACY-1215 was chosen as the MTC. (C) 50 and 100 µM of NF2373 was lethal to the larvae. 25 µM of NF2373 was safe without any adverse effects and determined to be the MTC. Representative larval images are shown on the right.

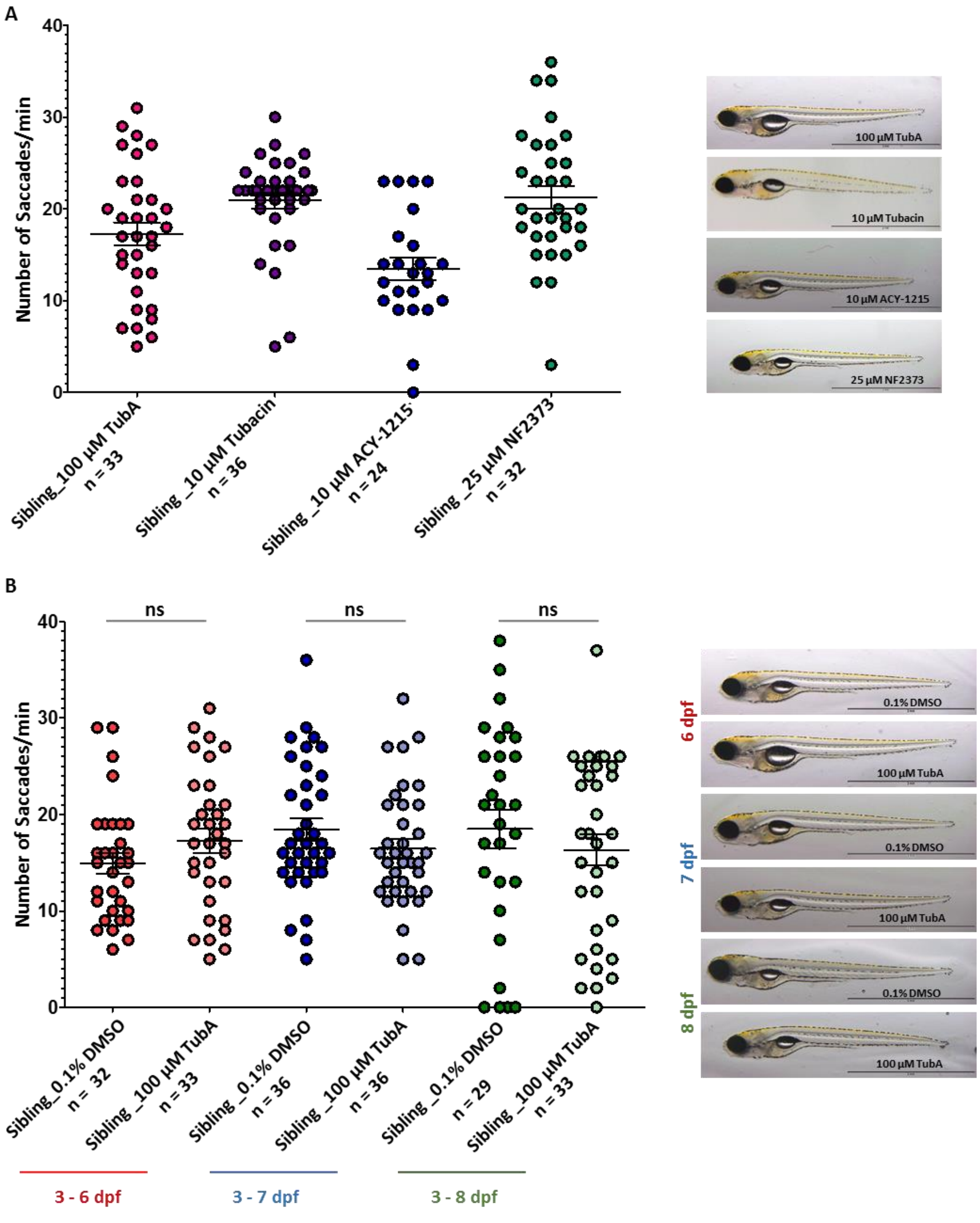

Supplementary Figure 2: OKR data of siblings for treatment conditions. (A - B) Siblings from *atp6v0e1* line were used as internal controls for the HDAC6i treatment conditions (N = 3 and n = 12/treatment group). Representative larval images are presented on the right.

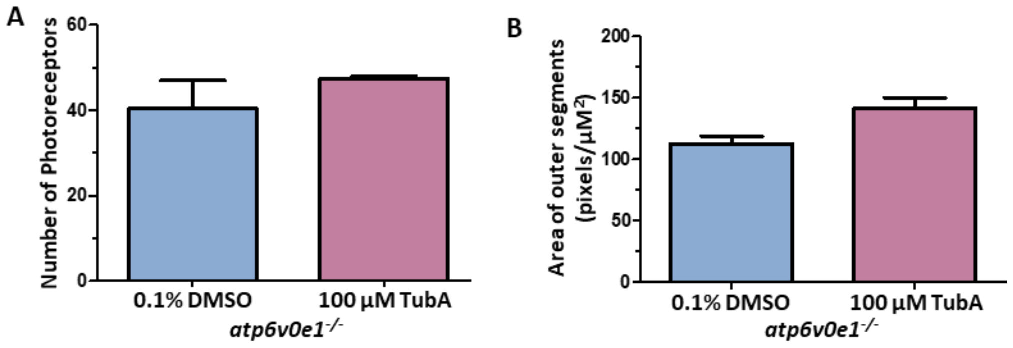

Supplementary Figure 3: EM analysis of photoreceptor number and outer segment area in *atp6v0e1* mutant retina treated with Tubastatin A. (A) TubA treatment did not result in a marked difference in the number of photoreceptors in *atp6v0e1*$^{-/-}$ compared to vehicle-treated *atp6v0e1*$^{-/-}$. (B) EM analysis suggests an increase in outer segment area of 25.7% in *atp6v0e1* mutants following TubA treatment, but this has not been demonstrated to be statistically significant (3 sections from the central retina per sample were counted from EM images, N = 2).

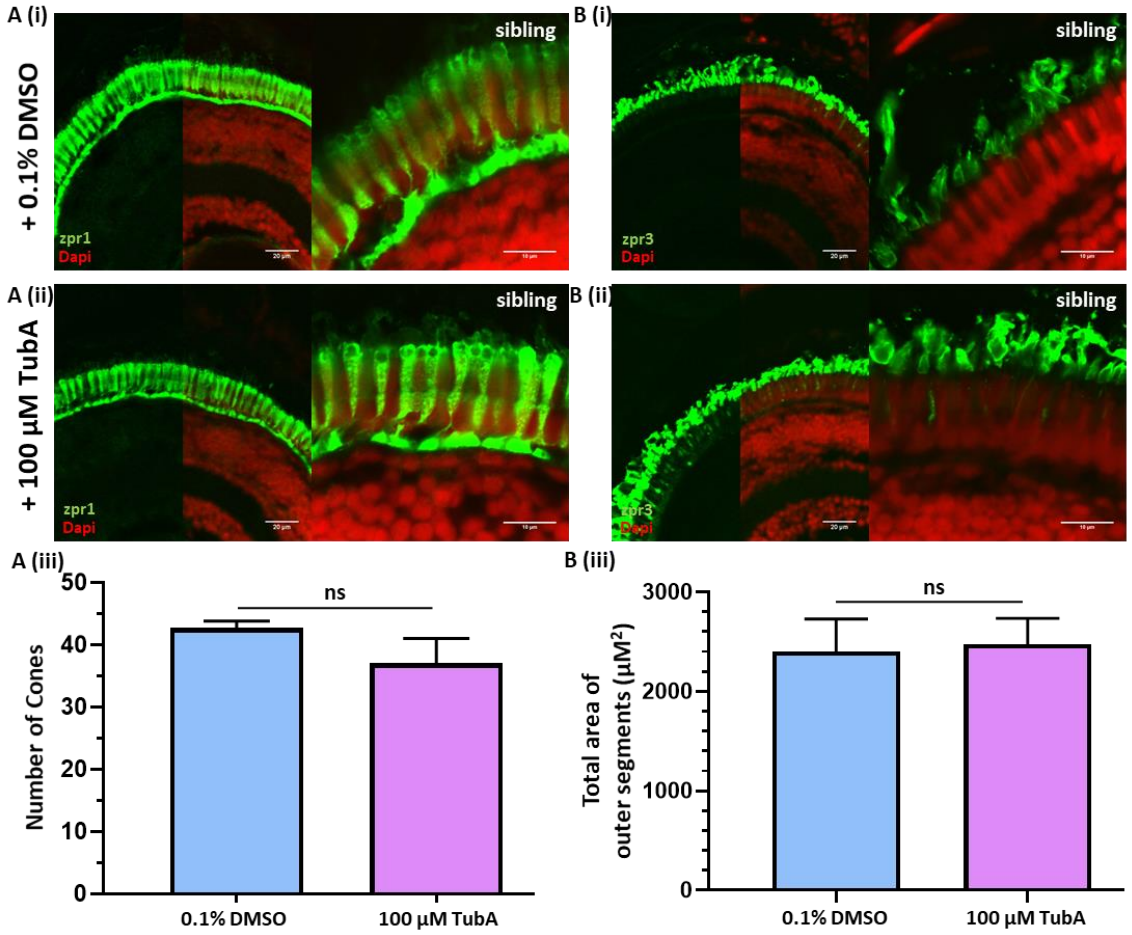

Supplementary Figure 4: Immunohistochemical analysis of retina from siblings treated with Tubastatin A. (A - B) Siblings from *atp6v0e1* line were used for analysis of cone and rod photoreceptors histology. Representative retinal images are presented. Graphical presentation of number of cone cells (zpr-1 staining) and total area of photoreceptor outer segments (zpr-3 staining), respectively (3 sections per sample was analyzed, N = 3). Student's unpaired T-test was used for statistical analysis.
